# Plasma bioscience and its application to medicine

**DOI:** 10.1007/s43673-021-00012-5

**Published:** 2021-04-07

**Authors:** Eun H. Choi, Han S. Uhm, Nagendra K. Kaushik

**Affiliations:** 1 Plasma Bioscience Research Center/Applied Plasma Medicine Center, Department of Electrical and Biological Physics, Kwangwoon University, Seoul, 01897 Republic of Korea; 2 Canode # 702, 136-11 Tojeong-ro, Mapo-gu, Seoul, 04081 Republic of Korea

**Keywords:** Nonthermal plasma, Biocompatible gas plasma, Plasma medicine, Health and hygiene, Clinical trials, Plasma commercialization

## Abstract

Nonthermal atmospheric pressure biocompatible plasma (NBP), alternatively called bio-cold plasma, is a partially ionized gas that consists of charged particles, neutral atoms and molecules, photons, an electric field, and heat. Recently, nonthermal plasma-based technology has been applied to bioscience, medicine, agriculture, food processing, and safety. Various plasma device configurations and electrode layouts has fast-tracked plasma applications in the treatment of biological and material surfaces. The NBP action mechanism may be related to the synergy of plasma constituents, such as ultraviolet radiation or a reactive species. Recently, plasma has been used in the inactivation of viruses and resistant microbes, such as fungal cells, bacteria, spores, and biofilms made by microbes. It has also been used to heal wounds, coagulate blood, degrade pollutants, functionalize material surfaces, kill cancers, and for dental applications. This review provides an outline of NBP devices and their applications in bioscience and medicine. We also discuss the role of plasma-activated liquids in biological applications, such as cancer treatments and agriculture. The individual adaptation of plasma to meet specific medical requirements necessitates real-time monitoring of both the plasma performance and the target that is treated and will provide a new paradigm of plasma-based therapeutic clinical systems.

## Introduction

Nonthermal biocompatible plasma (NBP), alternatively called nonthermal atmospheric pressure plasma (NAPP) [1–9] or cold atmospheric pressure plasma (CAP) [10–26], has been widely used in many areas of life sciences, such as plasma medicine [24, 27–34], agriculture [35–46], and disinfection against microbial bacteria and fungi [47–57]. This is especially prevalent now for disinfection against viruses, such as SARS-COV-2 (COVID-19) [58–63]. NBP may also be used for semiconductor technologies for large-area surface cleaning processes [64] and the removal of contaminated thin film for next-generation system semiconductor technology [65]. There are also many reports on plasma food technologies, such as germination growth and the germicidal activity of bacteria, fungi, and viruses [66–77].

Disinfection of microbials using NBP may be achieved through interactions between the plasma reactive oxygen or nitrogen species and soft materials in cell membrane protein, RNA, or DNA. Most plasmas are generated in the gaseous state using air, helium, neon, argon, nitrogen molecules, and their mixtures, according to the required purpose. Moreover, plasma-treated water (PTW), or plasma-activated water (PAW), may be employed for applications similar to those of gaseous plasmas without any challenges [78–85]. In this paper, we may refer to PTW or PAW as appropriate to the context. Direct contact between the target surface and plasma or the indirect treatment of PTW on the substrate causes the inactivation of bacteria and viruses with better efficiency and a faster rate than conventional methods. Additionally, plasma agriculture applications have been rapidly grown during the last decade to enhance seed germination, pathogen-related resistance of plants and seeds, the storage of harvested products, and horticulture; the applications also extend to food safety issues.

The purpose of this review is to provide a general survey of plasma bioscience and medicine technologies and their recent results for health and hygiene applications. Throughout this review, various NBP sources will be surveyed, and their characteristics will be briefly introduced. Among the many plasma sources, most NBP plasma sources are dielectric barriered plasma jets, dielectric barriered surfaces, or facing discharged plasma. Their driving frequencies may be low (less than 1 kHz), medium (ranging from ~ 10–50 kHz), or high (from radio ~MHz frequencies up to microwave GHz frequencies). These NBP plasmas may be characterized into one category because their electron density ranges from ~ 10^12^ to ~ 10^16^ cm^− 3^, which is beyond that of glow discharge plasmas. However, their electron temperatures range from ~ 0.8 to ~ 3 eV, which is quite similar to those of normal glow plasmas. For electrical medical equipment applications, the electrical leakage currents or forwarding currents to patients’ skin should be less than 100 μA for electrical safety according to IEC60601–1. The plasma gas temperature in plasma plumes is also very important because patients cannot be subject to heat above 45 °C. The hazardous gases, especially ozone O_3_, should be kept at a concentration of less than 0.05 ppm during 8 h of working operations according to the international and domestic regulations made by the Korean government (CFR801.415 maximum acceptable level of ozone: KS C9314).

This review presents many studies that were broadly performed in the field of plasma biosciences and medicine based on the convergence of multidisciplinary sciences, such as plasma physics, chemistry, biology, medicine, dentistry, and material sciences, particularly for NBP interactions with water or biological materials. The fundamental topics of investigation arising from these NBP interactions with water or biomaterials are reactive oxygen species (ROS) and reactive nitrogen species (RNS) that are generated by ambient air molecules during plasma discharges.

**Fig. 1:**
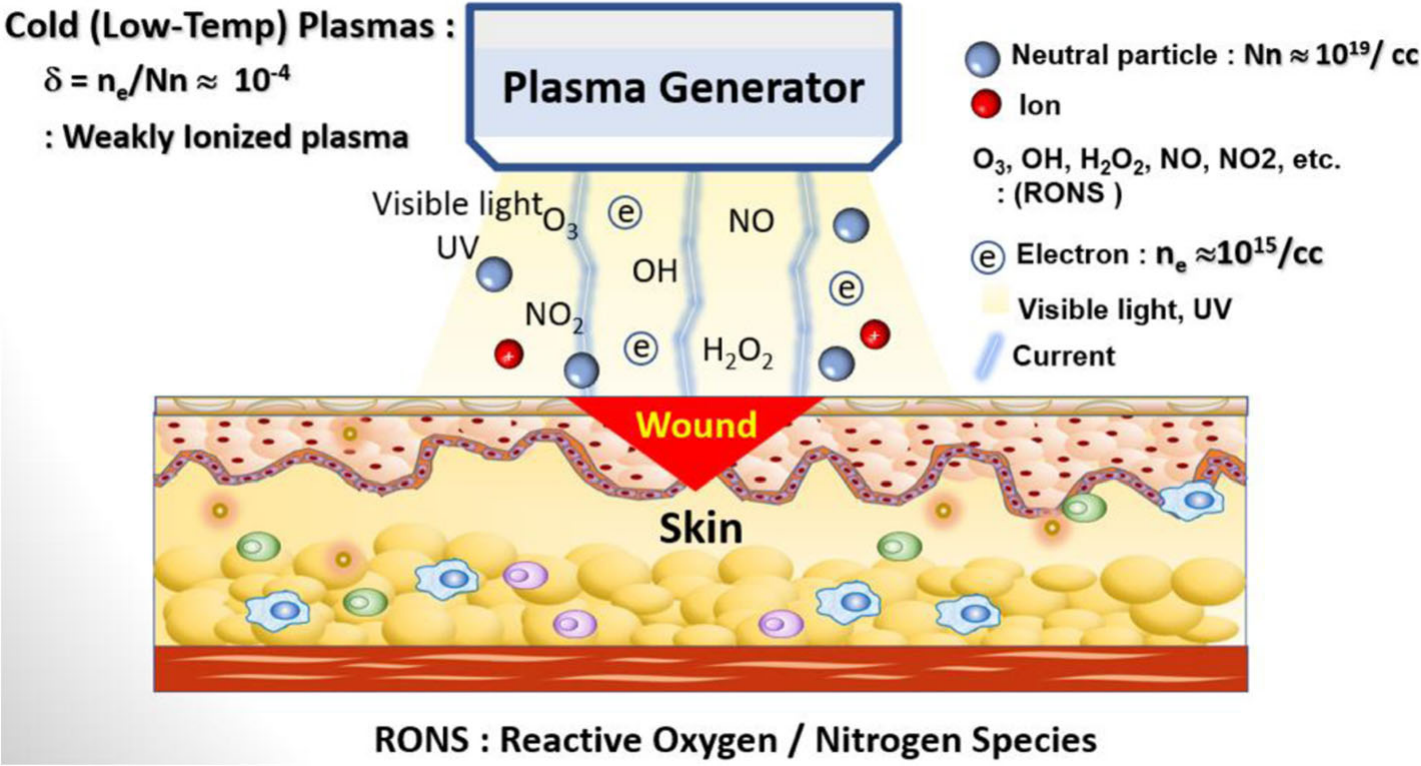
Schematic of reactive oxygen/nitrogen species (RONS) interactions with biological tissue. The RONS are generated from NBP at an ambient atmospheric pressure

Figure 1 shows the schematic of a basic mechanism that demonstrates how RONS, such as O_3_, OH•, H_2_O_2_, NO, NO_2_, and ultraviolet (UV), which coexist in NBP, may be generated and delivered to water or biological tissues during plasma interactions, even though plasma electrons and ions cannot propagate directly into skin layers. Here, we introduce the fast process of plasma-initiated UV photolysis and the slow diffusion processes for RONS to penetrate into water and tissue for plasma biosciences and medicine. Their synergistical interactions may enhance RONS penetration into water or cells in the tissue of biological systems for health care and medicine. Recent investigations of plasma biosciences and medicine are reviewed, with a focus on NBP sources, their plasma diagnostics, and their applications to plasma biosciences and medicine, such as agriculture, dentistry, cancer treatment, wound healing, and virus inactivation treatment.

## Plasma sources

### Nonthermal biocompatible gas plasma: basic physics and chemistry

Plasmas do not naturally exist on the earth’s surface. Therefore, required plasmas must be made via electrical breakdown. Electrons in a space with an electric field *E* collide with neutrals, which ionizes and generates new electrons, where the ionization constant *α* described by electrical field *E* and gas pressure *p* is expressed as [86]

1 $$ \upalpha \left(\frac{E}{p}\right)= hp\exp \left(-\frac{gp}{E}\right), $$

where *h* is the ionization cross section, and *g* represents the unique property of the gas. The ionization constant *α* represents the number of ionizing collisions made on average by an electron as it travels one centimeter in the direction of the electric field. Considering the increase *dn* of electrons over a distance *dx* and the number *n* of electrons crossing a plane a distance *x* away from the cathode per second, we may write *dn = αndx*. The solution to this formula is *n*(*x*) = *n*_0_exp(*αx)*, where *n*_0_ is the number of electrons per second leaving the cathode. The number of electrons increases exponentially as they propagate from cathode to anode. The ionization constant *αiE/p*) generally increases as the electric field *E* increases or the gas pressure *p* decreases.

**Fig. 2:**
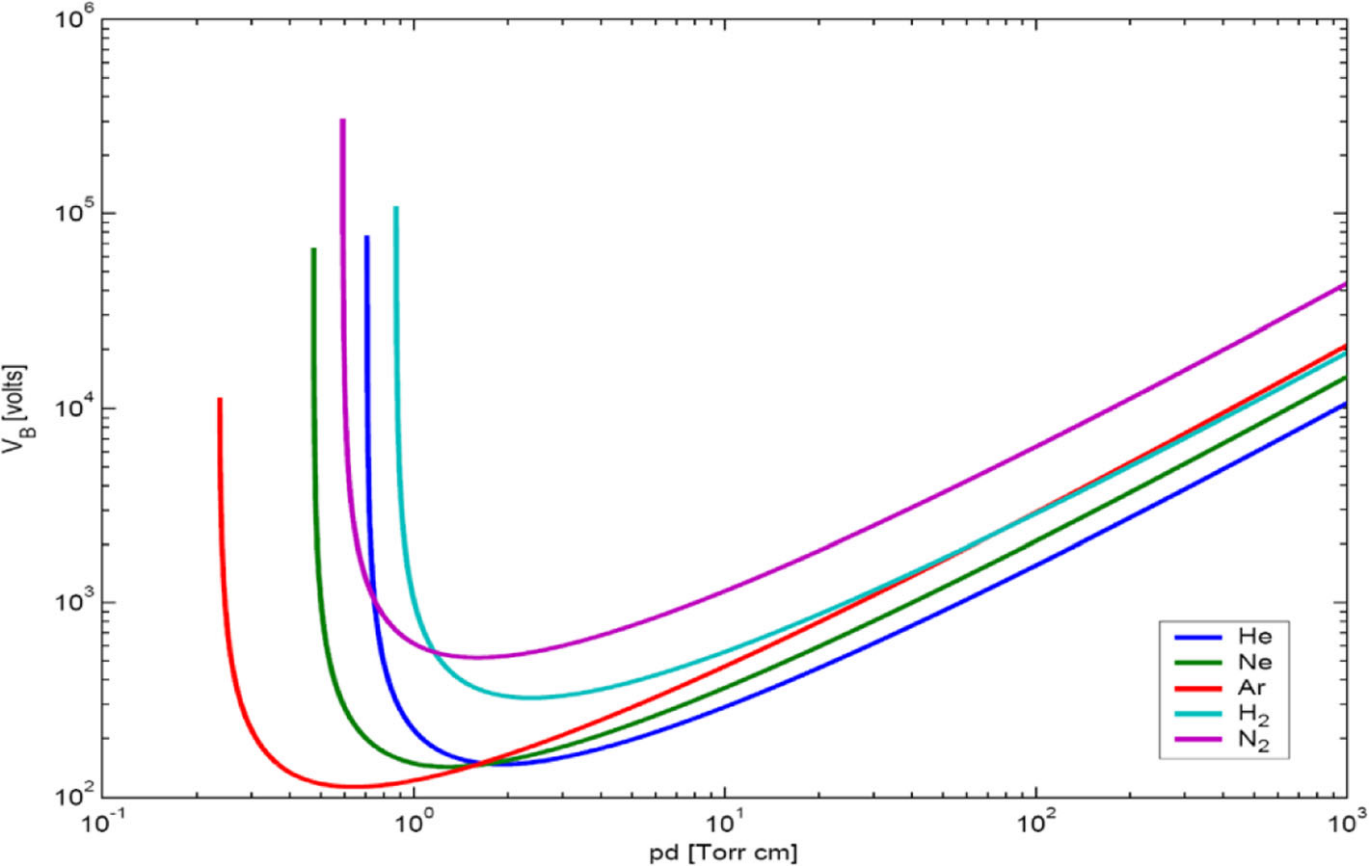
Paschen curves for the breakdown voltages for different gases in terms of *pd* in units of Torr.cm

The breakdown property of a gas in the space between a cathode and anode with a distance *d* is given by *α*(*E*/*p*)*d* = ln(1 + 1/*γ*), where *γ* represents the secondary electron emission coefficient, which indicates the number of electron emissions from the cathode whenever one ion falls to the cathode. Substituting the ionization constant *α*(*E*/*p*) into the gas breakdown property, the breakdown voltage defined by *V*_*B*_ = *Ed* is expressed as [86],

2 $$ {V}_B(pd)=\frac{gpd}{\ln \left[\frac{hpd}{\ln \left(1+\frac{1}{\upgamma}\right)}\right]}, $$

where the breakdown voltage is a function of gas pressure *p* and the distance *d* between electrodes. The breakdown voltage may be different for different gases, owing to their unique property of the constant *g*. Figure 2 presents plots of the breakdown voltage for a few gases which are called Paschen curves for the breakdown voltage.

Several points are noteworthy from Fig. 2. First, the breakdown voltage is a function of *pd*. Second, the breakdown voltage increase as the pressure *p* increases, owing to the increased collisions between electrons and neutrals. Third, the breakdown voltage increases if the value of *pd* is too small. The electrons arrive at the anode without collision if there are not many neutrals. Fourth, each gas has its minimum breakdown voltage *V*_M_ [86]

3 $$ \mathrm{VM}= gph=2.72\left(g/h\right)\ln \left(1+1/\upgamma \right)\ \mathrm{at}\  pd=\left(2.72/h\right)\ln \left(1+1/\upgamma \right). $$

Electrons transmit along the direction of the electric field *E*; they accelerate and gain more energy, but eventually collide with neutrals. The mean free-path *λ* of electrons in a molecular environment is the average distance without collisions with neutrals. Clearly, the mean free-path is inversely proportional to the gas pressure *p*. The mean free-path decreases as the collisional cross section of gas molecules increases. The energy gain of electrons increases as the electric field intensity increases, and the mean free-path becomes longer. Therefore, the electrons gain more energy at a lower pressure. This view point is the basis of plasma generation at atmospheric pressure. The electron temperature *T*_*e*_ is related to the electric field *E* and the mean free-path as *T*_*e*_ = *ξλeE*, where *e* is the electron charge, and *ξ* is the form factor of gas [87]. Then, the ionization constant can be expressed as [87]

4 $$ \upalpha \left({T}_{\mathrm{e}}\right)=2{n}_0q\upnu {\upvarepsilon}_i\exp \left(-\frac{\upvarepsilon_i}{T_{\mathrm{e}}}\right), $$

where *n*_0_ is the density of neutrals, *q* is the increase rate of the ionization cross section, *νi* is the collisional frequency of the electrons, and *ε*_*i*_ is the ionization potential of the neutrals. The mean free-path *λ* is inversely proportional to the neutral density *n*_0_ and electron scattering cross section *σ*, which is thereby expressed as 1/*λ* = *n*_0_*σ*. Recognizing all of these definitions, we note that the ionization constant defined by the electric field *E* is the same as that defined by the electron temperature *T*_e_.

### Nonthermal biocompatible plasma source operating at atmospheric pressure

Plasma sources applied at atmospheric pressure can be classified into thermal plasmas and nonthermal plasmas. The former’s plasma electron temperatures are nearly equal to surrounding ions and neutral particles via complete or local thermal equilibrium, whereas the latter’s plasma electrons are not in thermal equilibrium with their ions and neutral particles. This latter plasma is called a nonthermal, or cold, partially ionized atmospheric pressure plasma, which can be classified into direct and indirect plasmas. The electrons, ions, and RONS from the direct plasma were totally bombarded on a target (or samples) contained in a well plate (or dielectric barrier materials) because this target was placed between powered and grounded electrodes. However, for an indirect plasma source, the RONS are the main plasma species that bombard the target, instead of ions or electrons, because the target is placed out of the grounded electrode. Most electrons and ions generated from an indirect plasma flow to the grounded electrode via a dielectric barrier material, in which they are capacitively coupled to each other. Hence, most plasmas used for plasma bioscience and medicine belong to nonthermal dielectric barrier discharged (DBD) plasma, which are generated either by a direct or indirect method at an ambient atmospheric pressure. They may be driven by various different frequency ranges, including medium frequency, radio frequency, and microwave frequency.

The nonthermal atmospheric pressure plasma sources developed in the medium frequency range are typically plasma jets or long plasma tubes, where the frequency of the power supply is in the order of tens of kilohertz or less. Nonthermal biocompatible plasma jets (NBPJs) operating at ambient atmospheric pressure have been widely used among plasma devices. NBPJ devices are capable of generating nonthermal plasmas at atmospheric pressure without direct electrical contact to the liquid [88, 89]. The main operational features of NBPJs are as follows: (1) the ionized gas from the plasma jet exits through the nozzle, from where it is directed to the substrate and can be utilized in downstream processing; (2) NBPJs produce a stable, homogenous, and uniform discharge at atmospheric pressure; (3) the gas temperature of the discharge is low, which allows it to treat delicate surfaces without damage; (4) it may be operated by the dielectric barrier discharge between the electrodes; hence, it is free from filaments, streamers, and arcing. The reactive species are convectively transported to the liquid produced by NBPJs through the gas flow. NBPJs are typically operated as dielectric barrier discharges with a central power needle electrode [90], single-electrode jets with capacitive coupling [91], or either one or two outer ring electrodes [92, 93]. Excitation frequencies of NBPJs are typically in the form of a continuous wave or modulated waves [94]. In many NBPJs, the plasma is in the form of in linear jets, where the electric field is parallel to the gas flow, and the plasma will be in direct contact with the liquid if the discharge is sufficiently close to the liquid. However, the cross-field jets (the electric field is generally perpendicular to the gas flow) are not typically in electrical contact with the liquid, and the plasma-liquid interaction is dominated by neutral species [95]. Norberg et al. showed that a visible electron-ion plasma plume that touched the water surface was dominated in terms of spreading the reactive species because it had a direct charge exchange with water and direct solvation of electrons, thereby enabling plasma photolysis. As the treatment time increased, more aqueous ions and photolysis products were formed, whereas in non-touching plasma, the gas-phase ion fluxes produce the ion-ion plume, but it is smaller in magnitude compared with the direct touching plume. Additionally, the transport of these neutral species to the water surface in non-touching plasma is more sensitive to fluid dynamics [96].

A plasma source that generates a medium-range frequency was initially motivated by an economic sense because it is inexpensive and more convenient than alternative devices. Needle injection plasma [97] was first generated, for which an alternative current (AC) high voltage transformer provided power to the injection needle inside a Pyrex tube, and a working gas passed through the injection needle. A long plasma column, up to 40 cm, was generated inside the tube as well as a short plasma jet from the tip of the tube into the atmosphere. A cold plasma jet, made of a syringe needle covered by a glass tube, was later studied [98]. A long plasma column in a flexible tube at atmospheric pressure was generated [99], where the plasma system consisted of a typical injection needle used as a hot electrode, a Teflon tube used as a dielectric, and a high voltage power supply of 20 kHz. The plasma column was stabilized in the Teflon tube using the flow of argon gas through an injection needle. The column had a length of approximately 60 cm with 3 lpm of argon, and the plasma existed throughout the Teflon tube with an inner diameter of 1.6 mm.

Subsequently, twin injection-needle plasmas at atmospheric pressure were introduced [100] as a low-temperature nonequilibrium plasma source. Plasmas with long plasma columns of approximately 55 cm were produced from one AC power supply; however, they exhibited different characteristics, such as plasma column length and gas temperature. The twin plasma columns were regarded as thin rods with a uniform charge distribution, and a theoretical model was presented to investigate the change in the plasma column lengths with different distances between the plasmas compared with the change of the capacitance of the rods.

**Fig. 3:**
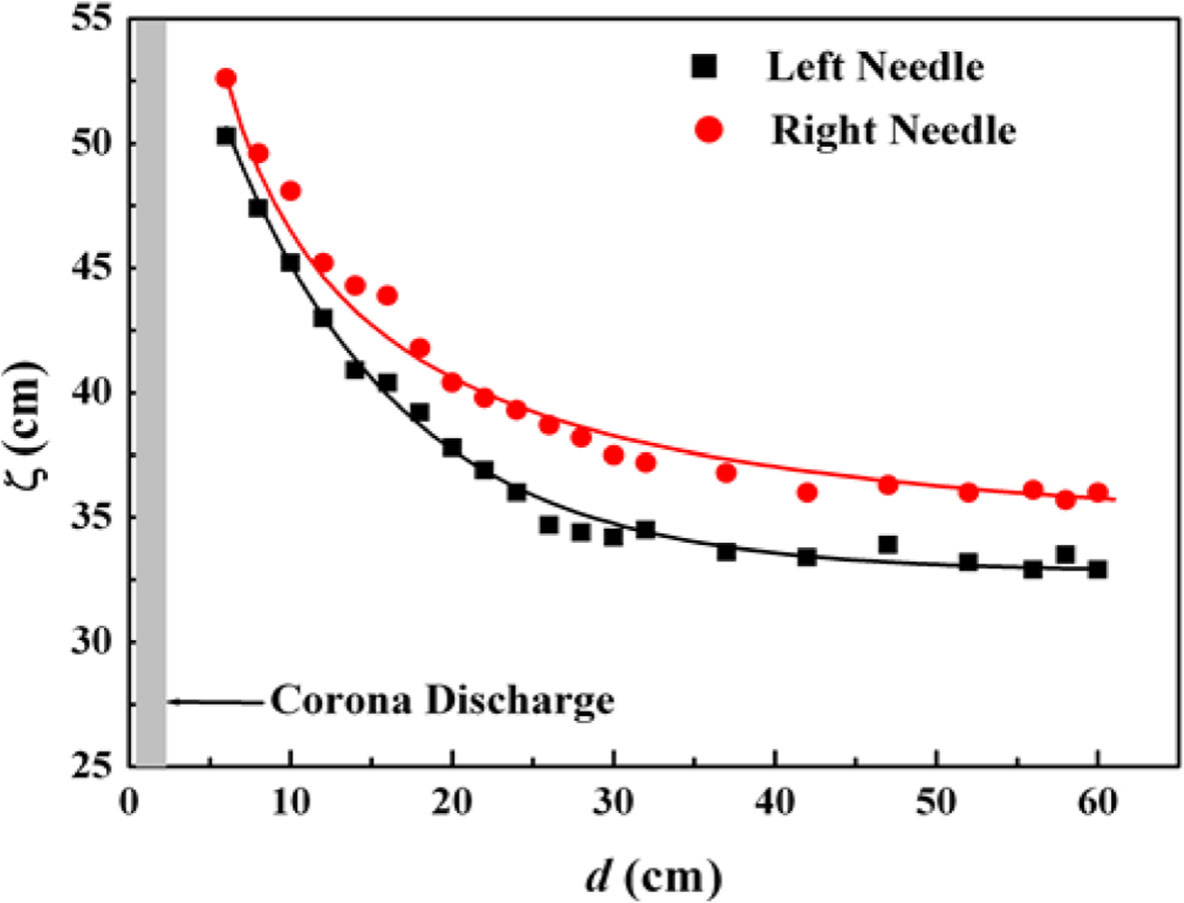
Plot of the column length (ζ) of injection-needle plasmas versus the distance (*d*) between the needles [89]

Figure 3 shows a plot of the plasma column lengths ζ for the left and the right needle dependent on the distance *d* between the needles. Experimental data for the left (square dots) and right (circular dots) needle plasmas are presented in Fig. 3 for a distance *d* in the range of 6–60 cm. The plasma column lengths in the range of *d* = 2–6 cm are not shown in Fig. 3. The lengths increased as the distance *d* increased because the dissipated energy of the corona discharge between two plasma needles was reduced. The maximum plasma column lengths occurred at *d* = 6 cm under those experimental conditions. The effects of the distance *d* in the range of 6–60 cm on the plasma column length are shown in Fig. 3. The total charge accumulated on the plasma column was proportional to the capacitance *C*(*d*), which is a decreasing function of *d*. The energy *W* per unit length stored in the plasma column is given by *W* = (1/2)(*C/d*)*V*^2^, which may be used for gas discharge in the column. The plasma column volume is proportional to the column length for a specified radius of the capillary tube and proportional to the energy *W*, thereby being proportional to the capacitance *C(d)* for a constant voltage *V*. Therefore, the column length is proportional to the capacitance *C(d)*, i.e., ζ = *κC(d).* The solid lines represent the theoretical results, which have been fitted to the measurement data using a least-squares fit. The plasma column length in Fig. 2 at a distance in the range of 30 cm < *d* < 60 cm is almost independent of the distance, as predicted from theory.

Two injection-needle plasmas were later developed into electric-shock free plasma jets [101], which were applied to bacteria [102]. A plasma jet without an electrical shock was generated through a Y-shaped tube, in which voltages with opposite phases were applied to a pair of tubes. The plasma plume generated at the intersection had a plasma potential of 60–90 V and high concentrations of reactive species that were sufficient to induce a high level of lethality on gram-negative bacteria on a tissue mimic. The selective lethality of bacteria on an epithelial-cell-containing tissue mimic may be modulated using oxidant and antioxidant chemicals, thereby leading to the possibility of a shock-reduced plasma jet for biomedical applications.

The plasma column generated in a glass tube was further studied to investigate the influence of the gas flow Reynolds number on plasma [103]. Atmospheric plasma generation inside a glass tube is influenced by gas stream behavior, as described by the Reynolds number (Rn). In experiments with He, Ne, and Ar, the plasma column length increased with an increase in the gas flow rate under laminar flow, which was characterized by Rn < 2000. The length of the plasma column decreased as the flow rate increased in the transition region of 2000 < Rn < 4000. For a turbulent flow beyond Rn > 4000, the length of the plasma column was short in front of the electrode, which eventually led to a shutdown. The propagation of plasma diffusion waves in a plasma column generated inside a glass tube was further studied [104] and optically measured [105].

**Fig. 4:**
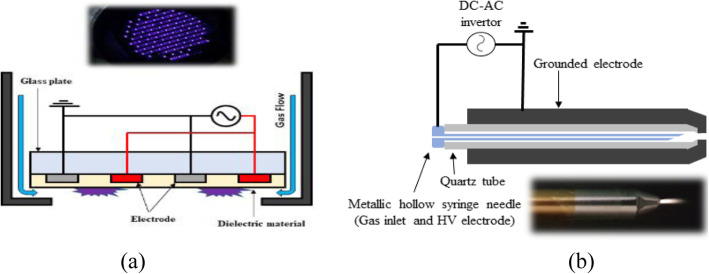
Schematic presentation of the atmospheric pressure air- (or nitrogen-) plasma jet device with a quartz dielectric tube. The inset in **a** is a photograph of the 5 lpm air- (or nitrogen-) plasma jet and surface dielectric barrier discharged plasma (S-DBD). The inset in **b** is a photograph of the 2 lpm air- (or nitrogen-) plasma jet [107]

The first biologically applicable plasma jet was the air plasma jet with hollow electrodes [106] at atmospheric pressure; the jet device was operated by injecting pressurized air into the electrode hole. A genuine air or nitrogen plasma jet for biological application was developed by operating a 10–100-kHz high-voltage inverter driving system with a duty cycle adjustment for many applications [14] or a continuous 60 Hz AC transformer. A schematic of the air- (or nitrogen-) plasma jet device with a quartz dielectric tube is shown in Fig. 4a. The plasma jet system primarily consisted of a power needle electrode, quartz tube, grounded electrode, and a high-voltage power supply. The quartz tube served as a guide for the plasma jet flow and was designed to induce dielectric discharge through the sheet current along the concave part at the end of the quartz tube between the grounded electrode and the needle. The outer electrode was fabricated from stainless steel and had a somewhat conical shape; it was centrally perforated with a 1-mm hole, through which the plasma jet was ejected to the surrounding ambient air. The distance between the needle tip and the grounded electrode was 2 mm, and the plasma jet plume was produced approximately 5 mm from nozzle. Micro-discharges along the quartz tube were ejected as a plasma jet from the outer electrode through a 1 mm hole, which showed that the temperature of the jet decreased to a value close to the ambient temperature [14, 15]. Soft plasma jet discharge was achieved using a DC-alternating current (AC) inverter. The air (or nitrogen) plasma jet device with an average power less than 5 W exhibited a cold plasma jet with an approximate length of 2 cm near the ambient temperature, which is low enough to treat thermally sensitive materials. A plasma jet may be generated using noble Ar gas, which provides the benefit of producing long plasma plumes with a gas flow rate of more than 3 lpm. Preliminary studies on the discharge characteristics indicated that the electron temperature was 1.56 eV, and the plasma density was approximately 1.8 × 10^12^ cm^− 3^. A microplasma jet was also generated at atmospheric pressure for nitrogen gas [108], where the measured gas temperature of the jet was 290 K, which was the ambient temperature. Observation of striated multilayer discharge patterns that formed in the plasma jet [109] was conducted using nitrogen plasma. The jet device in the micro-hollow electrode had a pencil-type configuration that produced a long cold plasma jet, which was capable of reaching 3.5 cm, and various excited plasma species were shown through the optical emission spectrum. By introducing a gas flow rate of more than 5 lpm, striated discharge patterns in the plasma jet were produced through ionization wave propagation.

Many types of dielectric barrier discharge (DBD) plasma source have been developed since it was first investigated by Siemens in 1857. DBD, which is also called silent discharge or barrier discharge, is a specific type of AC discharge, which provides strong thermodynamic nonequilibrium atmospheric pressure plasma at moderate gas temperatures, which are seen in plasma display panels. DBD plasma sources can be manufactured using several geometrical configurations, such as dielectrically separated parallel plate structures and coplanar surface discharged plasma types. The electrical discharge can be produced and sustained by two electrodes, such that each electrode applied using alternating high voltage is masked by a dielectric layer placed in the current path between the metal electrodes. The dielectric walls are commonly made of glass, bakelite, quartz, ceramics, and polymers. Energetic electrons are formed in the discharge region, owing to the ionization process. These electrons can collide with neutral gas atoms, such as nitrogen, oxygen, and water vapor, and dissociate them. The distance between the electrodes can be varied from less than 0.1 mm to several centimeters. As shown in Fig. 4b, one of the fundamental DBD plasmas is a coplanar surface dielectric barrier discharged plasma (S-DBD). This may be used for the generation of facing DBD discharged plasma by facing two coplanar DBD panels opposite to each other according to their applications. An AC voltage with an amplitude of 1–10 kV and a frequency from line frequency to several MHz is applied to DBD configurations. DBD plasma can be produced in various working mediums through ionization by high frequency and high voltage electric discharge [110].

**Fig. 5:**
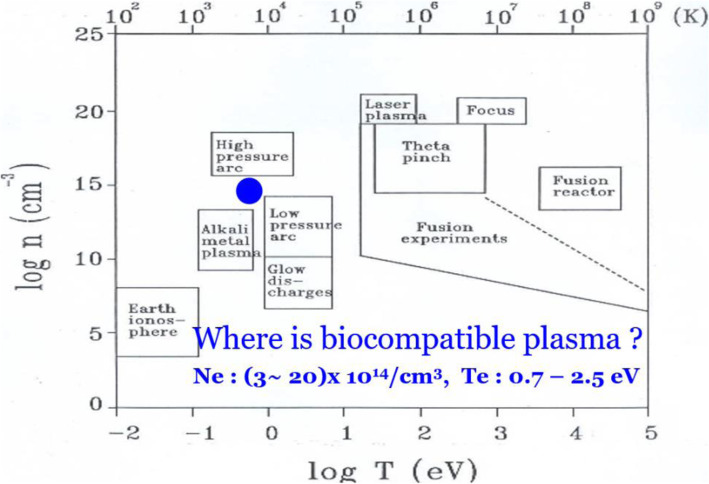
Characteristics of nonthermal atmospheric pressure plasma sources for plasma bioscience and medicine [111]

Figure 5 shows the characteristics of NBP sources that are being used in plasma bioscience and medicine. These sources are operated at atmospheric pressure and may also be used for material processing applications and surface modifications of polymers [111]. The electron densities *n* and temperatures *T* of NBP plasma have been measured and estimated to be 3–20) × 10^14^ cm^− 3^ and 0.7–2.5 eV, respectively [111], using various plasma diagnostics. The plasma electron density for NBP can typically be well described by Stark broadening and laser optical interferometry methods. Additionally, the electron temperature for NBP can be measured using the atmospheric pressure collisional radiative method from Ar excited 2p spectra and the wave packet ambipolar diffusion method based on the two-step ionization model. The gas temperature from NBP plasma can be measured by rotational N_2_ SPS emission spectra N_2_(A_3_Σ_u_^+^) [111]. This plasma can generate various types of radicals when it comes into contact with water molecules. Hydroxyl radical species have a particularly important role in the biological and chemical decontamination of media in this situation. In this context, hydroxyl radical density was measured [112]. A needle-typed plasma jet bombarded the water surface using an Ar gas flow, and the emission lines by OES were investigated. It was noted that the electron temperature and plasma density were measured to be approximately 1.7 eV and 3.4 × 10^14^ cm^− 3^, respectively, under an Ar gas flow that ranged from 80 to 300 sccm in this experiment [112]. The hydroxyl radical density OH• has also been investigated and measured at a maximum value of 2.6 × 10^15^ cm^− 3^ for a gas flow rate of 150 sccm in the needle-typed plasma jet using UV optical absorption spectroscopy. The hydroxyl radical density OH• inside a bio-solution was measured [113] using UV absorption spectroscopy when a plasma jet was bombarded onto the solution. The emission and absorption profiles for the other reactive oxygen species, such as NO (226 nm) and O_2_*- (245 nm), were very small inside the bio-solution in comparison with those for the OH radical species. The densities of OH• radical species inside the bio solutions were higher than those on the surface in this experiment. The densities of the OH• radical species inside the deionized water, Dulbecco’s modified Eagle’s medium, and phosphate-buffered saline were measured to be approximately 2.1 × 10^16^, 1.1 × 10^16^, and 1.0 × 10^16^ cm^− 3^, respectively, 2 mm downstream from the surface under an optimized Ar gas flow of 200 sccm. Additionally, the critical hydroxyl OH radical density for lung cancer H460 cells to experience an apoptosis was observed to be approximately 0.3 × 10^16^ cm^− 3^ under 1 min plasma exposure in this experiment. The OH• radical density in the vicinity of the water surface depends very sensitively on the gap distance between the plasma jet nozzle and liquid surface. The average OH• concentration reduced from 6.1 × 10^15^ cm^− 3^ to 1.3 × 10^15^ cm^− 3^ as the gap distance increased from 1 to 4 mm [114].

The hydroxyl radical OH• is highly reactive and damages all types of macromolecules in cells; thus, it has been suggested that it is a major cause of biological effects. However, there remain many unknown questions about its lifetime, propagation, and interaction with cells in aqueous solutions. Visualizing the highly reactive OH radical interactions with cells inside a solution can be achieved by utilizing the deuterium isotope. Heavy water (D_2_O) containing the deuterium isotope was introduced into a plasma jet device to generate deuterium monoxide (OD•) radicals instead of hydroxyl (OH•) radicals [115, 116]. *Escherichia coli* (*E. coli*) in water was treated with OD• radicals, and D atom incorporation into cells was visualized using time-of-flight SIMS and nano-SIMS [115]. The results show that D atoms from the plasma jet reach the cytoplasm of *E. coli* in H_2_O, indicating the usefulness of this OD•-tracking method for the study of radical interactions with living cells.

As previously mentioned, one of the most important radicals produced from a plasma jet is the hydroxyl OH•, which can be most efficiently produced from nitrogen plasma. Thus, generation of various radicals in nitrogen plasma and their behavior in media was investigated [117]. Research on the generation of radicals in nitrogen plasma shows that the most dominant radicals are excited nitrogen molecules in the metastable state of N_2_(A_3_Σ_u_^+^). Hydroxyl molecules are generated from the dissociation of water molecules upon contact with excited nitrogen molecules. The behavior of these radicals in media was investigated. Excited nitrogen molecules in the N_2_(A_3_Σ_u_^+^) state from a plasma jet were injected into water, after which the molecules disappeared within a few tens of nanometers, thereby producing hydroxyl molecules. Hydrogen peroxide, hydrogen dioxide, and nitrogen monoxide molecules can diffuse much deeper into water, which implies the possibility that a chemical reaction between hydrogen dioxide and nitrogen monoxide molecules produces hydroxyl molecules in deep water, even though the density in this case may not be very high.

**Fig. 6:**
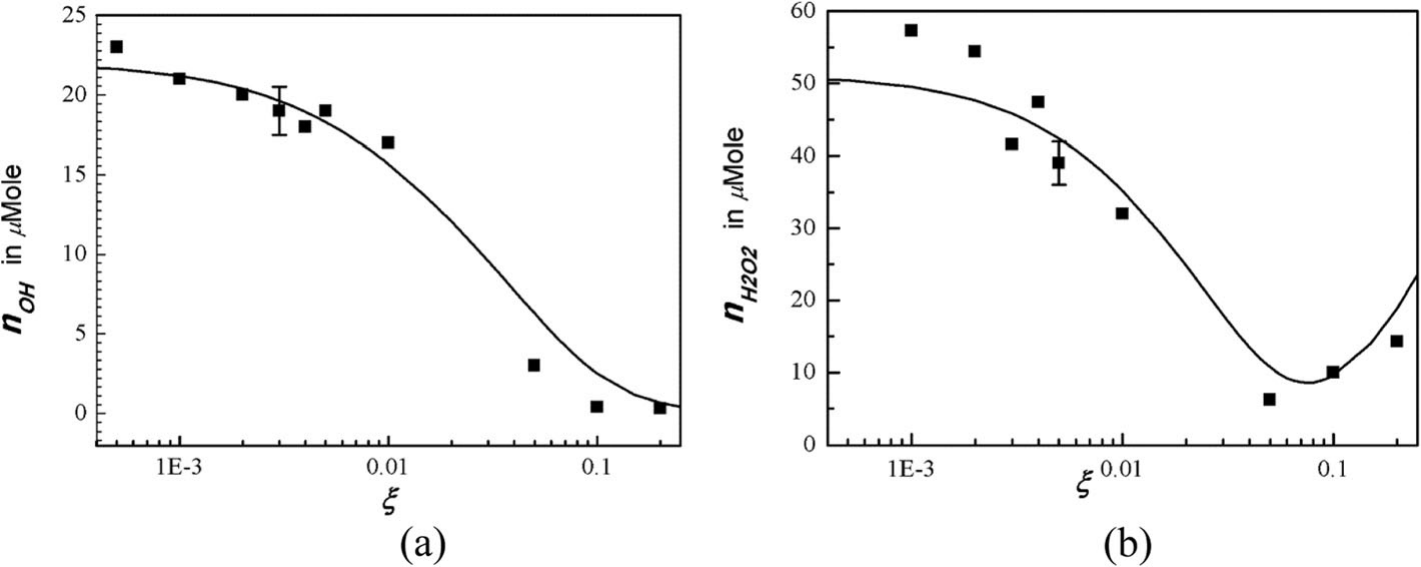
**a** Hydroxyl OH• density in deionized water activated by a nitrogen plasma jet versus the oxygen mole fraction of *ξ*. **b** experimental data (dots) of hydrogen peroxide versus the oxygen mole fraction *ξ* [103]

Nitrogen plasma alone may produce a specified radical at prescribed conditions. Thus, the influence of oxygen on the generation of reactive chemicals from a nitrogen plasma jet was investigated [118]. Various chemical compounds were fabricated from nitrogen and water molecules in a plasma jet with a varying oxygen content. A detailed theoretical investigation of these chemical compounds was conducted for different oxygen ratios *ξ*. Experimental measurements were also conducted for a comparison with theoretical results. Hydroxyl molecules were mostly generated at the surface of water, and some of them penetrated into the water. The density of hydroxyl molecules reached its maximum without oxygen, as shown in Fig. 6a, and decreased to zero as *ξ* increased to 0.25. The density of the ammonia of NH_3_ also decreased as *ξ* increased to 0.25. However, both the theory and experiment demonstrated that the density of the NO_3_ increased significantly as *ξ* increased to 0.25. The hydrogen peroxide density in plasma-activated water decreased, reached its minimum value at *ξ* = 0.05, and increased again as *ξ* increased from a small to a large value, as shown in Fig. 6b. The pH value of the plasma-activated water, which is slightly alkali without oxygen, decreased as *ξ* increased.

### Plasma discharges on liquid and its chemistry

**Fig. 7:**
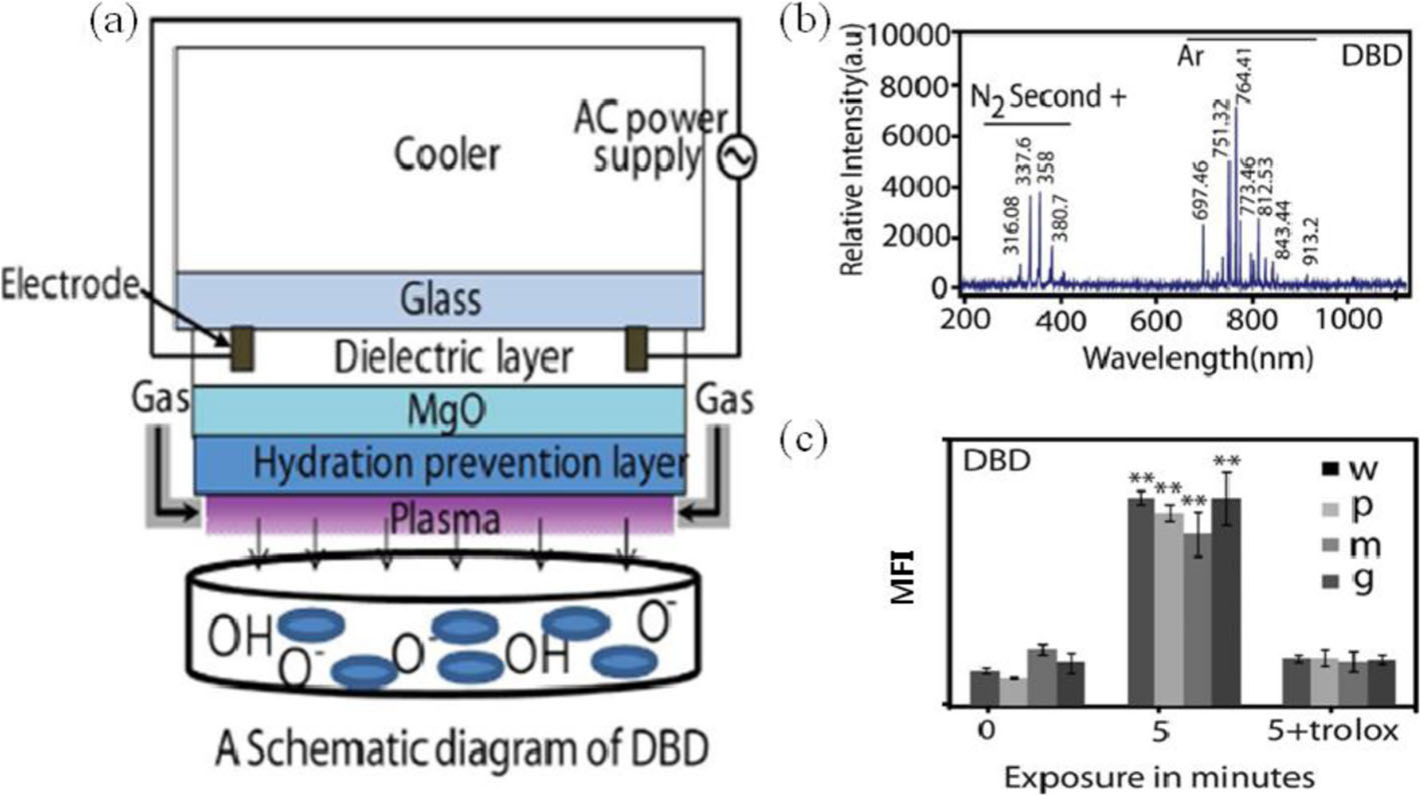
**a** Schematic diagram of a coplanar Ar S-DBD, **b** its optical emission spectra, and **c** its intracellular ROS estimation after a coplanar Ar S-DBD treatment against super-bacteria with the presence of an ROS scavenger Trolox. All values in **c** are expressed as MFI and ± standard deviation in triplicates. Students’ *t* test was performed as a control (* denotes *P* < 0.05 and ** denotes *P* < 0.01) [119]

To generate plasma discharges on a liquid or water, surface discharged or coplanar S-DBD plasma may be used with a duty-ratio controlled sinusoidal pulse generator. Figure 7 shows (a) a schematic diagram of a coplanar Ar S-DBD plasma device, (b) its emission spectra, and (c) the intracellular ROS estimation after a coplanar Ar S-DBD treatment along with the presence of an ROS scavenger Trolox [119]. It is noted that there are plasma-generated UV lines with wavelengths ranging from 210 to 400 nm. These are responsible for plasma-initiated UV photolysis inside water, cell, or tissue, and they exist for coplanar S-DBD plasma. This S-DBD plasma device has been fabricated using photolithography technology based on plasma display semiconductor technology. Its electrodes had a width of 100 μm, and they were placed in parallel on the same plane with a 100 μm gap distance. Additionally, these coplanar electrodes were coated by SiO_2_ dielectric materials with a 70-μm thickness for low voltage operation at less than 1 kV. This was followed by a hydration prevention layer with a 1-μm thickness.

The coplanar S-DBD plasma has been used for the treatment of S. aureus bacteria (wild-type:w) and multidrug-resistant bacteria, such as Penicillium-resistant S. aureus (PRSA:p), methicillin-resistant S. aureus (MRSA:m), and gentamicin-resistant S. aureus (GRSA:g), and the viability of all bacteria was checked. It was observed that the bacteria were inactivated by a logarithmic order of 4–5, and the scanning electron microscopy images show the crushed and ruptured surfaces of S. aureus after the plasma treatment. The Ar-DBD plasma has a maximum intracellular ROS for a treatment time of 5 min. These intracellular ROS may be produced synergistically by plasma-initiated UV photolysis inside the cell along with extracellular ROS transport into the cell. It was also concluded that reactive species play an important role in the inactivation of bacteria to a large extent [119]. Owing to an increase in extracellular ROS or stress, the intracellular ROS also increased. The intracellular ROS for Ar-DBD increased by 3 times, relative to the control sample. It could be predicted that extracellular ROS or RNS radicals, which have either a short or long lifetime, play a crucial role in increasing the intracellular ROS of the multidrug-resistant and wild-type *S. aureus* bacteria, owing to stress. However, less intracellular stress was observed in the presence of Trolox, as shown in Fig. 7.

### Water as a UV photolysis layer during plasma cell-tissue treatment

During plasma discharge, the ions, electrons, neutral particles, and UV radiation contribute to the generation of RONS in the gas and liquid phase. Vacuum UV (VUV) radiation (10–200 nm) can efficiently induce reactions in liquid, particularly near the gas-liquid interface [120]. The absorption coefficient of water (liquid state) is 10^3^–10^5^ cm^− 1^ at *λ* < 170 nm [120], which leads to an absorption of ~ 90% of the radiation by a thin water layer (10^− 3^–10^− 5^ cm); however, at *λ* ≈ 185 nm, the penetration depth is approximately 0.1 cm [121]. It was also determined that VUV radiation on water dissolved with O_2_ results in the formation of OH•, H, hydrated electrons, and O (singlet and excited state) [122–124]. A study by Jablonowski et al. investigated the VUV radiation generated by an “kINPen09” atmospheric pressure Ar plasma jet [125]. They observed that the VUV range was controlled by the second Ar excimer continuum at *λ* ≈ 126 nm. The adsorption spectra of VUV was measured using a deuterium lamp in different liquids, such as distilled water, sodium chloride solution, and Rosewell Park Memorial Institute (RPMI 1640) cell culture media.

**Fig. 8:**
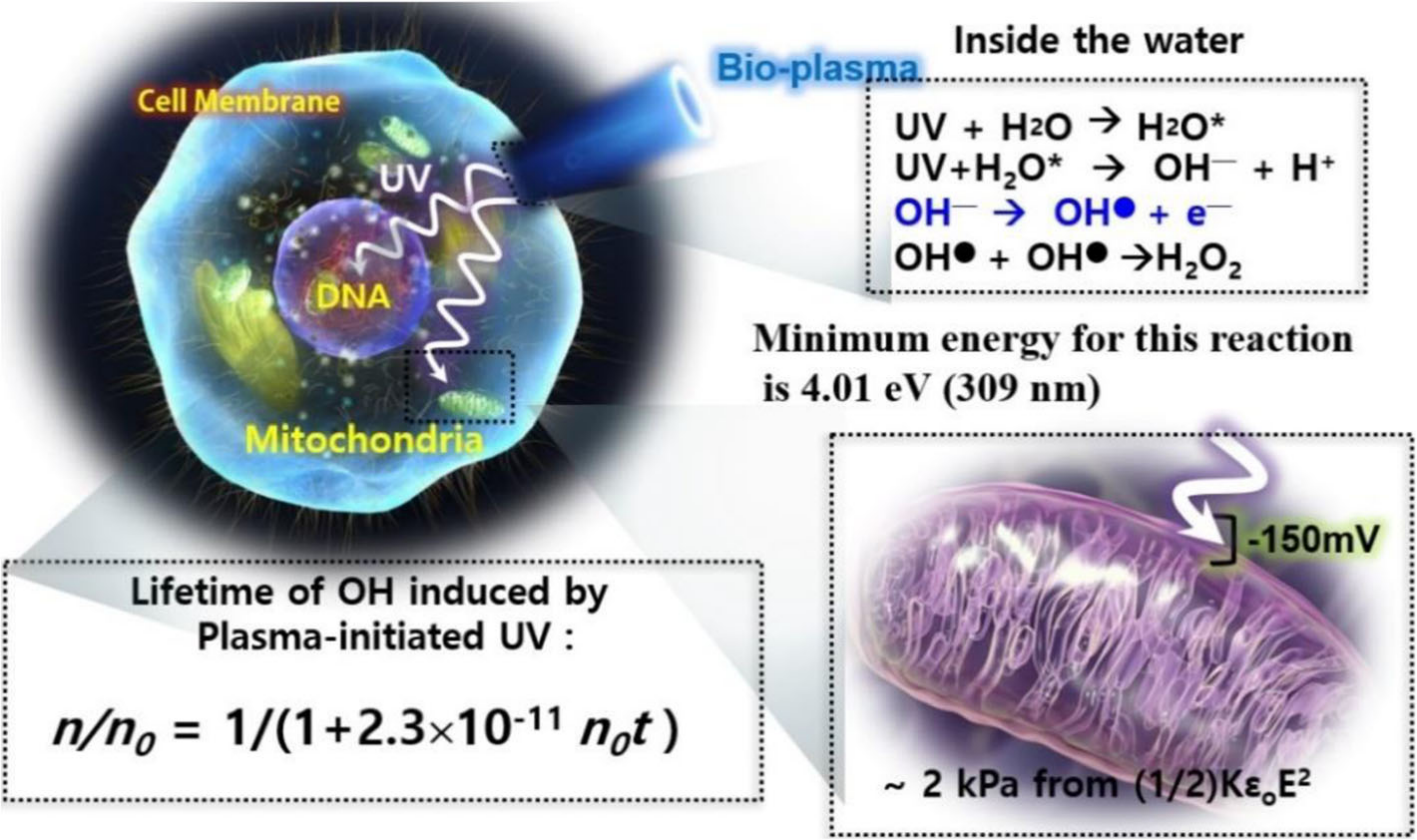
Plasma-initiated UV photolysis: generation mechanism of reactive oxygen species inside a cell or biological tissue [111]

The lowest cutoff wavelength among all liquids was observed for water at ~ 180 nm, depending on the layer thickness. As the complex solution increased, the cutoff wavelength shifted higher; the cutoff wavelength for RPMI 1640 was between 210 and 230 nm. Additionally, the concentration of H_2_O_2_ formed because the complete plasma jet treatment on H_2_O was more than two orders of magnitude higher than that formed by VUV radiation of the plasma jet. Finally, they concluded that it becomes impossible for VUV radiation to reach the cells when they are covered by a thin liquid layer of at least 25 μm. Therefore, the authors concluded that VUV radiation emitted by a plasma jet has a negligible effect in plasma medicine [125]. UV radiation with *λ* < 185 nm penetrates into the aqueous bulk solution, and the photochemical reactions may occur with molecules of water and other naturally occurring organic compounds, nitrates, and nitrites [126]. Previous studies have focused on the plasma-initiated UV photolysis that may be accountable for the simultaneous and continuous generation of OH• species in a liquid or inside a cell [127], as shown in Fig. 8. Subsequently, NBPJ was used with argon as a feeding gas and treat water [127, 128]. The quartz filter was used at the interfacial region between air and a water surface to screen the electrons, ions, and neutral particles generated by NBPJ; therefore, only plasma-initiated UV emitted radiation could pass through the filter for propagation into the water. The plasma-initiated UV emitted from the excited primary RONS near the water surface, the wavelegth of which ranged from ~ 200 to ~ 400 nm, may have interacted with water molecules to produce the OH• species, and consequently, to H_2_O_2_ during propagation into the water or interior region of the cell. The minimum energy of these reactions was approximately 4 eV, which corresponds to 309 nm. The OH• emission lines ~ 309 nm were investigated 2 mm, 4 mm, and 6 mm below the water surface.

**Fig. 9:**
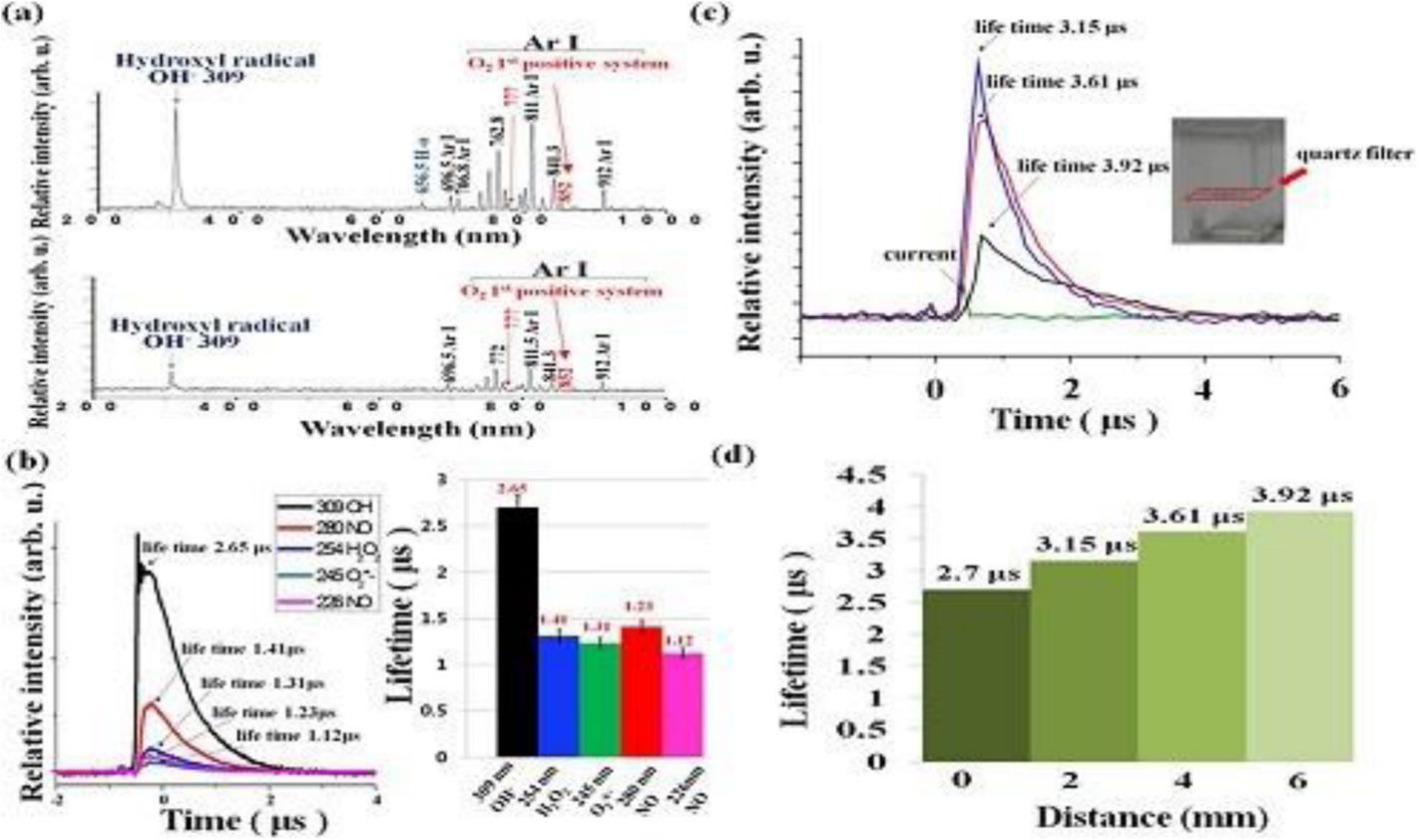
**a** Optical emission spectrum 2 mm above and below the surface of DI water. **b** Lifetimes for various ROS measured in the ambient air 2 mm above the water surface. **c** Temporal behaviors at 309 nm for OH emission intensity for different depths of 2 mm, 4 mm, and 6 mm in the DI solution with a quartz filter at a depth of 1 mm. **d** Lifetimes of OH• versus water depth positions of 2 mm, 4 mm, and 6 mm from the surface. Here, an Ar plasma jet has been bombarded onto the DI surface [127]

Figure 9 shows (a) the optical emission spectrum in the 2 mm region above (top) and below (down) deionized (DI) water and (b) lifetimes for various ROS measured in the ambient air region 2 mm above the water. The lifetime of OH• has been measured to be 2.7 μs on the surface. Figure 9c depicts the temporal behaviors at 309 nm for OH emission intensity for different depth locations, 2 mm, 4 mm, and 6 mm, inside the DI solution with a quartz filter at a 1 mm depth position in the water, and (d) shows the lifetimes of OH versus water depth positions of 2 mm, 4 mm, and 6 mm from the surface under Ar plasma jet bombardment on the water surface. These lifetimes are 3.15 μs, 3.61 μs, and 3.92 μs at 2, 4, and 6 mm below the water surface, respectively. For a specified depth of 2 mm in DI water, the temporal behavior of OH• emission lines (~ 309 nm) could be fitted to 1/(1 + 2.3 × 10^− 11^ *t*) based on the empirical measurements in this experiment. However, the density of OH• species decreased as the water depth increased; the density was 4.2 × 10^16^ cm^− 3^ at a 2-mm depth, but 8.0 × 10^15^ cm^− 3^ at a 6-mm depth. Moreover, the mitochondrial cell membrane pressure was reduced by 2 kPa from its typical value of − 150 mV when the OH• species generated by plasma UV photolysis with a concentration of ~ 3 × 10^16^ cm^− 3^ at a 2-mm depth was bombarded onto the mitochondrial cell membrane. In conclusion, it was found that plasma-initiated UV photolysis (energy ranged from ~ 4 to 6 eV) assisted in the production of various reactive species in water. Additionally, the following reactions were proposed for the formation of OH• [127, 128]:

5 $$ \mathrm{UV}+{\mathrm{H}}_2\mathrm{O}\to {\mathrm{H}}_2\mathrm{O}\ast $$

6 $$ \mathrm{UV}+{\mathrm{H}}_2\mathrm{O}\ast \to \mathrm{OH}\bullet +{\mathrm{H}}^{\bullet },\mathrm{or}\;\mathrm{O}+2\mathrm{H} $$

7 $$ \mathrm{OH}\bullet +\mathrm{O}\mathrm{H}\bullet \to {\mathrm{H}}_2{\mathrm{O}}_2,\mathrm{or}\;{\mathrm{H}}_2\mathrm{O}+\mathrm{O} $$

8 $$ {\mathrm{H}}_2\mathrm{O}\ast \to {\mathrm{OH}}^{-}+{\mathrm{H}}^{+} $$

9 $$ {\mathrm{O}\mathrm{H}}^{-}+{\mathrm{O}\mathrm{H}}^{-}\to {\mathrm{H}}_2{\mathrm{O}}_2+2{\mathrm{e}}^{-} $$

10 $$ {\mathrm{OH}}^{-}\to \mathrm{OH}\bullet +{\mathrm{e}}^{-} $$

11 $$ {\mathrm{O}}_3+{\mathrm{O}\mathrm{H}}^{-}+{\mathrm{H}}^{+}\to 2\;\mathrm{OH}\bullet +{\mathrm{O}}_2, $$

in which the reaction of Eq. (9), OH^─^ ➔ OH• + e^─^, may also play an important role in the formation of OH radicals because it requires very little energy (~ 2.4 eV), which may be the reason for OH formation in liquid.

It is necessary to investigate the RONS delivery via passive RONS transport from NBPJ throughout agarose to water versus the plasma UV photolysis from NBPJ throughout quartz to water, where the agarose can be used as an alternative to biological tissue [129–131]. An Ar NBPJ has been employed for DI water filled in a quartz cuvette. Interfacial water surface plays an important role in providing the plasma-generated primary RONS and other excited molecules, which can be monitored by UV-visible spectroscopy.

**Fig. 10:**
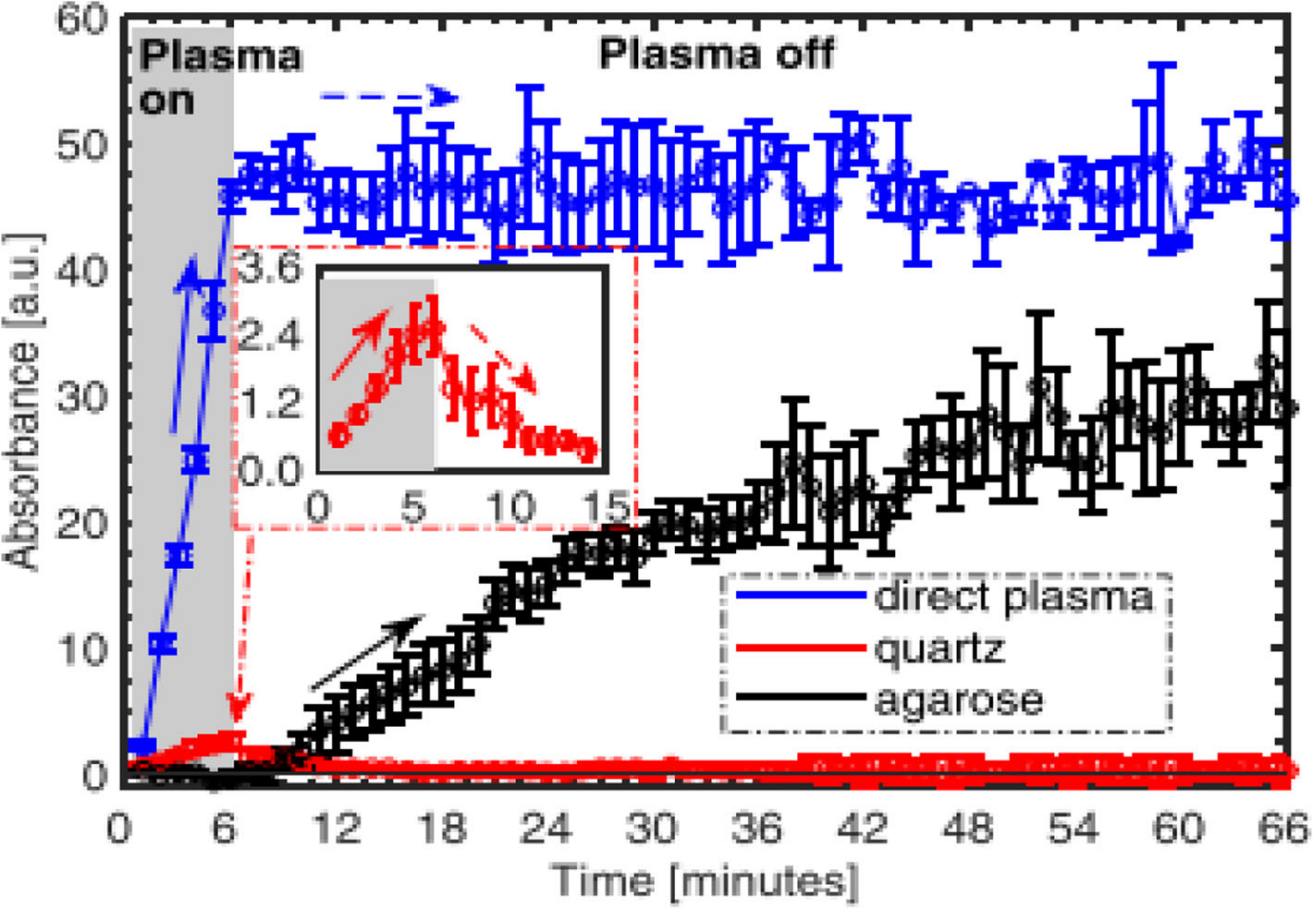
Time-dependent RONS delivery into water monitored using UV absorbance during and after direct exposure of Ar NBPJ and indirectly exposed through quartz for UV photolysis or through agarose for molecular transport. The shaded region denotes the time window during which the plasma is switched on (up to *t* = 6 min). At *t* = 6 min, the plasma and gas flow are turned off and RONS delivery measurements were acquired till *t* = 66 min. In the inset box, positive and negative slopes are denoted by solid and dashed arrows, respectively [132]

Figure 10 shows the time-dependent RONS delivery to DI water monitored using UV absorbance during and after direct exposure of Ar NBPJ on DI water. This figure shows the combined synergistic effect of molecular transport and plasma UV photolysis for RONS and the indirect exposure through quartz to observe the RONS delivery generated from plasma UV photolysis or through agarose with a thickness of 2.5 mm to assess the diffusion transport of RONS. The distance from the NBPJ plume end to the water surface was fixed to be almost 0 mm so that the plasma jet always makes contact with the water surface. The Ar NBPJ was switched on until *t* = 6 min and was subsequently turned off, as shown in Fig. 8. For the direct interaction of NBPJ with the water surface, a rapid and steep increase in the absorbance was observed since the plasma was switched on. Just after switching off the plasma and gas flow, the absorbance intensity remained saturated because additional RONS were not created. For the plasma-initiated UV photolysis treatment through the quartz plate, the total absorbance was observed to be a few percent compared with direct plasma bombardment. In this case, a relatively moderate concentration of the OH•, which was ~ 4.2 × 10^16^ cm^− 3^ at a 2 mm depth, was simultaneously generated inside the water by plasma UV photolysis. For the molecular transport of RONS through agarose when the plasma was switched on, till 6 min, the absorbance decreased to zero because the plasma UV was almost absorbed by the agarose. However, RONS dissolved into the agarose from the interfacial region between the ambient air and water surface started to undergo a diffusive transport process into water across the agarose layer. However, after the plasma was switched off after 6 min, the absorbance monotonically increased, and it was saturated beyond 60 min. This molecular delivery of RONS through agarose exceeded plasma UV photolysis, and the transportation of molecular RONS is a slow diffusion process. However, it is still emphasized that the molecular transport of RONS through agarose is smaller than that by direct plasma bombardment on water or tissue. Hence, the combined effects of plasma-initiated UV photolysis and molecular transport may be responsible for the RONS generation and delivery mechanism into a cell or tissue [132]. Moreover, other studies showed that He NBPJ could directly deliver RONS species of H_2_O_2_, NO_2_^−^, NO_3_^−^, and O_2_(aq) to DI water or tissue [133], where O_2_(aq) become the oxygen molecules in the water solution. Here, the molecular transport of O_2_(aq) into the tissue is known to be related to tissue oxygenation, which is opposed to oxidation.

### Influence of other factors on reactive oxygen and nitrogen species generation in DBD and NBPJ on/in water

RONS generation has also been affected by the electrical properties of plasma, electrical fields, humidity, and gas composition. The RONS concentrations can be controlled by output voltages, gas flow rate, and plasma exposures [134–136]. Moreover, the intensity of all RONS species in gas environments decreases for Ar-O_2_ plasma, compared with pure Ar plasma, because the addition of O_2_ decreases the number of electrons, owing to its electronegative behavior. In a liquid, it was observed that the concentrations of H_2_O_2_ and OH decrease for Ar-O_2_ and Ar-N_2_ plasma, compared with pure Ar plasma. However, the concentrations of NO_2_^−^ and NO_3_^−^ are high for Ar-N_2_ plasma, compared other plasmas [136]. He+O_2_ + H_2_O admixture plasma enables the generation of various ROS species, such as O, O_2_*, O_3_, OH•, and H_2_O_2_ [137]. Studies concluded that ROS produced by this plasma is unaffected by the ratios of H_2_O/O_2_ in the feeding gas. For a maximal simultaneous production of OH and O, the ratios of H_2_O/O_2_ in the feeding gas were ~ 1. However, it was also revealed that when the plasma plume was in contact with liquid, it generated a high concentration of H_2_O_2_, compared with the NO_2_^−^. Conversely, in the case where the plasma plume was not in contact with liquid, the concentration of NO_2_^−^ was high [138]. Furthermore, a comparison between the experimental and modeling results showed that reactive species detected in the jet are mostly formed in the gas phase inside the plasma jet, and the H_2_O_2_ concentration depends strongly on the humidity in the feeding gas, but not on the distance of the jet from the liquid surface [139].

This review presents various aspects of the generation of reactive species during plasma exposures on liquid. During discharge, the UV radiation plays the important role of generating the reactive species inside the cell or tissues. The concentration of the reactive species in the gas-phase, gas-liquid interface, and liquid bulk during plasma exposures on the liquid surface depends upon the distance between the plasma plume and liquid and plasma electrical parameters, including electric fields, humidity, gas mixtures, VUV, UV, and gas flow rate. Plasma-initiated UV photolysis [34, 127, 128] also has a significant impact on the generation of OH• radicals and other RONS below the plasma-liquid interface, intercellular regions, or inside the tissue. It is concluded that the combined effects of plasma-initiated UV photolysis and the transport of RONS into the cell or tissue may be responsible for the RONS generation mechanism inside the cell or tissue. The formation of OH• radicals in (or interfacial) regions of the water surface occurs in the following steps [34, 128, 132]. (a) RONS (OH•, NO, O_2_-, N2 ^+^) are produced in the vicinity of the water surface upon discharge initiation. These are primarily caused by electron impact, metastable argon/oxygen atoms, and nitrogen atoms. (b) Next, the RONS generated in the vicinity of the water surface initiates UV radiation, which excites the water molecules and produces OH• radicals below or on the water surface. (c) The primary RONS, which are generated by electron impact, and secondary RONS, by plasma-initiated UV photolysis, are both diffused into water according to their depths. Additional OH• radicals are produced through reactions with secondary RONS, such as HO_2_ and NO. These RONS are accumulated in the liquid surrounding the cells, and they consequently interact with the cellular membrane that protects the intracellular environment.

## Health and hygiene applications of nonthermal biocompatible plasmas

### Cancer treatment by plasmas

During the last several years, there is substantial progress in the development of cancer treatment approaches; many of them are yet high-priced and only available in advanced countries. Therefore, there is a need for inexpensive yet effective strategies for cancer treatment, however, particularly in less-economic countries, where a significant portion of the cancer-related deaths happen annually [140]. Recently, plasma-based therapy was developed as a new weapon that has been recognized as a safe as well as a cost-effective device to target a variety of cancers [10]. It has been evidenced that these atmospheric pressure gas plasmas can increase the efficiency of existing treatment methods when combined to improve their selectivity towards resistant cancers [29, 141].

#### Plasma-exposed treatment

Plasma treatment could be done by both ways of plasma exposure as well as non-exposure methods. In most cases, plasma exposure is widely used in a nonlethal manner to affect normal eukaryotic cell functions, by regulating cell signaling pathways [1], and lethally for the destruction of malignant tumors. Various researchers have been proved that a certain treatment dose of plasma can inhibit the growth of cancer cells in a selective manner using cell-line-based experiments against several cancer types [2, 10]. Micronucleus formation in cancer cells by plasma was studied [142], observing induction of micronucleus formation (cytogenetic damage) in brain cancer cells upon exposure of dielectric barrier discharge plasma. This article investigated the influence of exposure and incubation times on T98G brain cancer cells. It was found that the micronucleus formation rate directly depends on the plasma exposure time. The differential responses of six cancer cell lines after treatment of nonthermal plasma from dielectric barrier discharge (DBD) were studied [143]. Investigators described that plasma exposure leads to an induction of oxidative stress resulting in caspase activation, DNA damage, a mitochondrial impairment which eventually leads to cancer cell death. Moreover, Kaushik et al. suggested that the failure of the antioxidant system to neutralize ROS is responsible for ROS elevation and cell death in solid cancers. They showed that excess ROS and H_2_O_2_, which are not reduced by antioxidant enzymes inside the mitochondria, may also damage mitochondria and eventually cause cellular damage [144]. Also, to compare the plasma effect, they generated hydroxyl radical (HO·), superoxide anion (O_2_−), and H_2_O_2_ using chemical reagents inside an in vitro cell culture. These chemically generated ROS systems have a drastic effect on cell death induction in a dose-dependent manner which was not specific concerning the normal/or cancer cell type. They concluded that the accumulative effect of different reactive species generated by the plasma also affects normal counterparts, which have a less inhibitory effect than on cancer cells [3]. Another group of researchers showed that effect of direct treatment was comparable when used different types of plasma sources such as nanosecond pulsed dielectric barrier discharge (DBD) and a microsecond pulsed DBD jet when T-lymphoblastic cell lines were tested. Due to the induction of reactive species in culture medium, plasma can induce cytotoxicity in leukemia cells even when cultivated in hypoxic conditions, which plays a critical role in promoting chemoresistance [4]. Similarly, Haralambiev et al. suggested that no extensive differences in biological effects were observed in osteosarcoma cells when treated with two different plasma sources, MiniJet-R and kINPen MED. Regarding clinical use, it can be stated MiniJet-R, as well as the approved kINPen MED plasma source, appears to be suitable to be used as an anti-OS treatment therapy. However, animal tests are necessary to confirm whether these in vitro are detectable in clinical applications to be used in patient treatment [11].

Apart from the generation of chemical factors (highly reactive species), plasma also generates physical factors; UV radiation, thermal radiation, and electromagnetic (EM) waves [12, 145]. Recently, Keidar et al. showed that the EM waves emitted from plasma kill the melanoma cells through a transbarrier contactless technique. Interestingly, their method induces much stronger growth inhibition in a reactive species-resistant B16F10 melanoma cell. Such physically triggered growth inhibition is owing to the leakage of bulk solutions from the cells, causing cytoplasm shrinkage and cellular membrane bubbling on the cell membrane. These studies provide an insight into the use of plasma as a physically based non-invasive cancer treatment [13].

Reactive species and radiation produced by plasmas capable to deliver a range of consequences with relevance to oncology, including the selective killing of a wide range of cancer cells [31]. Such in the case of tumor excision, plasma technology can be used to decontaminate the malignant tumor site by treating remaining healthy tissues or removing pathogenic microbes, promoting new healthy tissue regeneration. The advantage of plasma treatment could be achieved by killing cancer cells directly that may have not been removed properly during excision, thus efficiently decreasing the tumor regrowth or metastasis. Clinically, physicians believe that direct plasma treatment is highly suitable for the treatment of primary tumors that arise from mucosal surfaces which can be considered as adjuvant therapy [146].

A new approach for cancer treatment was based on the congenital overproduction of hydroxyl radicals (OH•) and hydrogen peroxide (H_2_O_2_) in cancer cells. H_2_O vapor was applied as a synergistic agent to the plasma jet, which not only controls the temperature rise but also enhances selectivity by increasing the level of H_2_O_2_ and OH• radicals during treatment [147]. It was observed that due to the increased concentration of OH radicals, apoptosis was induced on SK-BR3 breast cancer cells through an enhancement of oxidative signaling, which inhibits the phosphorylation of extracellular signal-regulated kinases (Erks) and activates the phosphorylation of mitogen-activated protein kinases (p38-MAPK). The results showed that OH•/H_2_O_2_ plays a pivotal role in not only inducing cell death but also in enhancing the selective killing effect. This group also observed that it induced apoptosis on melanocytes G361 cancer cells through DNA damage signaling cascade. Additionally, it was observed that plasma induces ROS, which activated MAPK p38 and inhibits p42/p44 MAPK, leading to cancer cell death [148].

#### Immunomodulation and induction of immunogenic cell death

In the past, most of the cancer treatment approaches have focused on diminishing tumor burden through the delivery of cytotoxic agents via physical or chemical means. However, these methods usually do not rely on the individual’s immune responses for the insistence of cancer. Recently, cancer immunotherapy has recognized the utilization of oxidative stress-based methods to overcome cancers, called immunogenic cell death (ICD) induction in cancer cells. Consequently, emerging treatments that provoke ICD through the active participation of the patient’s adaptive immune system, offer the great potential to improve clinical outcomes of existing cancer treatment approaches. Since the anticancer consequences of plasma are largely endorsed through the reactive species that are generated or delivered inside the target cancer cells, it is quite reasonable to propose that plasma could be helpful for ICD induction. The advantage of plasma relies on its capability to enrich the interactions between plasma-exposed cancer cells and local immune cells which initiates successive protective immune responses for cancer treatment. Marian et al. emphasize the recent investigations of plasma application to induce ICD in cancer cells [149].

**Fig. 11:**
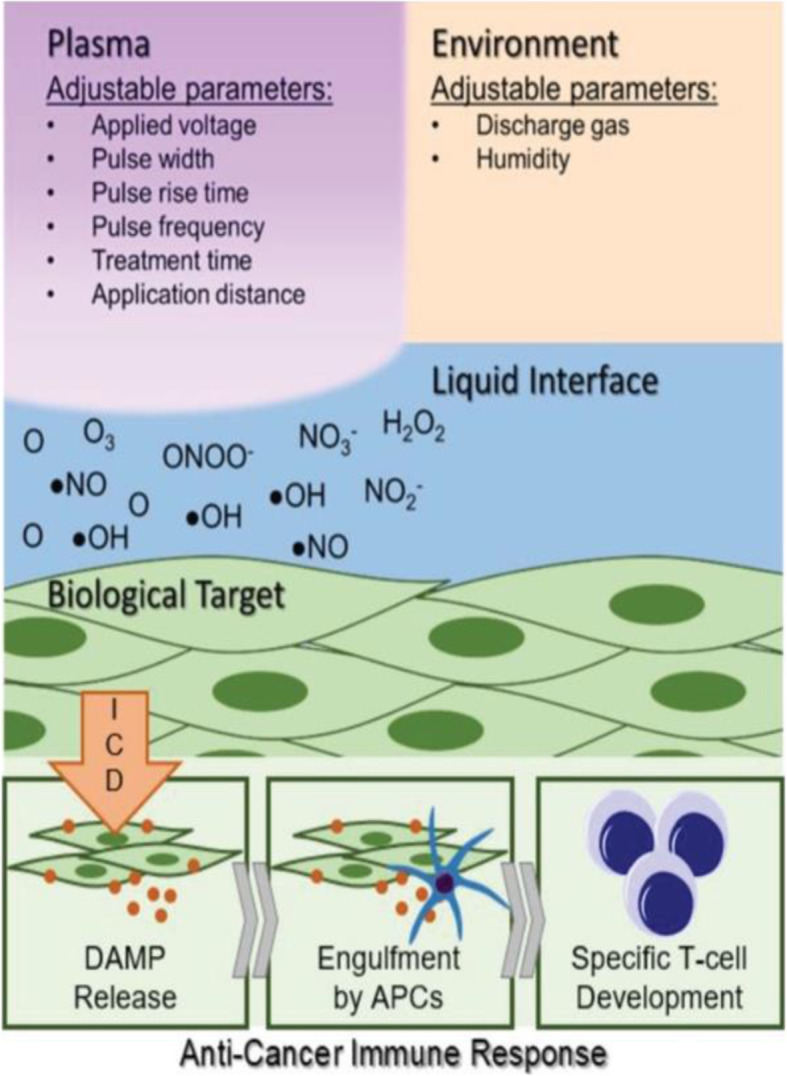
Understanding how nonthermal plasma interacts with cancerous targets would allow strategic development of a biomedical device for the controlled delivery of RONS to elicit immunogenic cell death. This could assist the initial steps of a patient’s cancer immunity cycle and lead to a robust anticancer immune response [8]

To understand further the role of plasma in ICD, this group further performed a detailed examination of the reactive species generated by plasma operated at ICD-inducing regimes. They claimed that not only persistent reactive species were adequate for plasma-induced ICD, also short-lived species plays were essential. Desired reactive species generated by the plasma device are entirely dependent on the plasma treatment energy and not on a specific treatment parameter such as application time, pulse frequency. Based on this study, it could be suggested that optimization of particular plasma parameters, would permit us to build-up a clinical tool for controlled delivery of reactive species required for ICD-based cancer therapy, as shown in Fig. 11 [8].

**Fig. 12:**
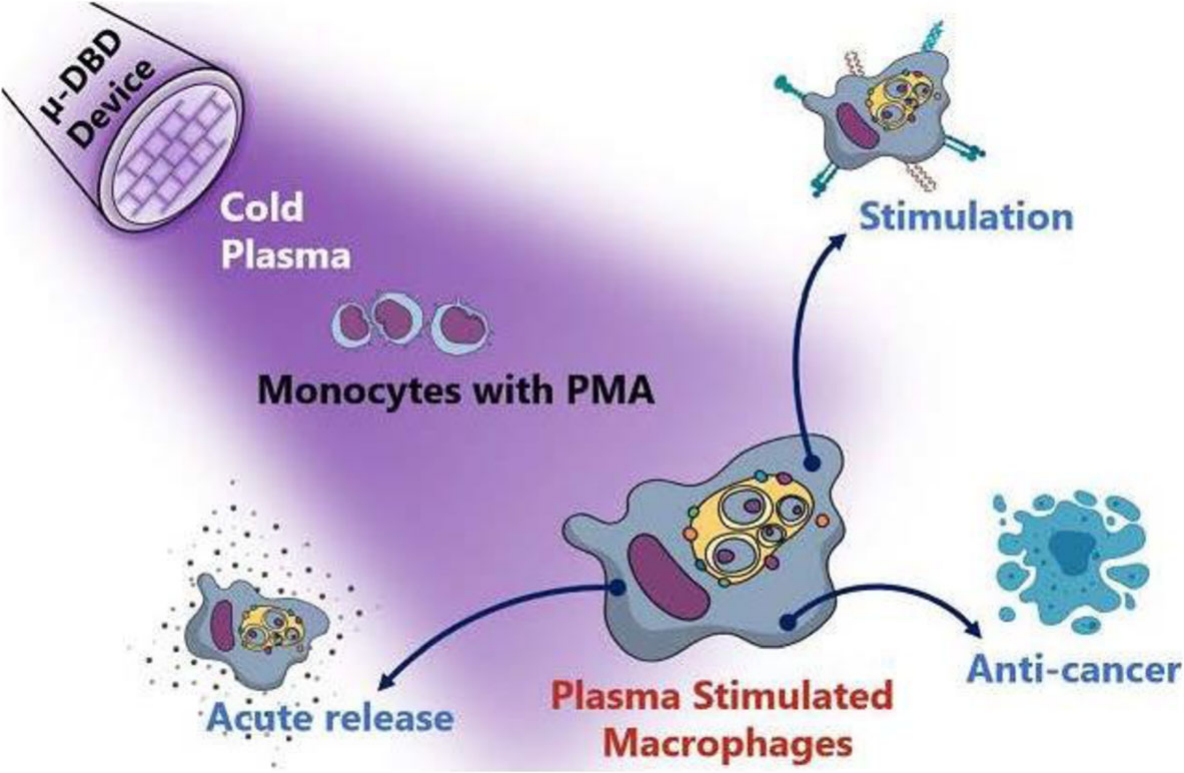
Plasma-induced immunomodulation: activation of monocyte/ macrophages [16]

Additionally, in recent times, there are several anticancer therapies designed to regulate the immune system widely [150] based on the use of cell-based therapies, cytokines, and immune checkpoint blockade (ICB). In this regard, strategies available to improve efficiency and minimize the side effects of ICB therapy are clinically significant. Taking this aspect into consideration, Guojun et al. described a method to use transdermal plasma for ICB therapy. Confined delivery of plasma via hollow-structured microneedles supports the discharge of tumor-associated antigens, ensuing in improved local and systemic anticancer immunity. The synergism between plasma and ICB combined with hollow-structured microneedles offers a platform system for various diseases including cancers [15]. Additionally, Kaushik et al. demonstrated that plasma can activate immune cells and inhibit the growth of solid cancers after co-culture through TNF-α release. Interestingly, they showed that plasma itself did not induce any cell death in RAW264.7 macrophages. These findings suggest plasma-generated reactive species have the potential to recruit cytotoxic macrophages to release TNF-α to provoke cancer cell death [151]. They further extended their study using a micro-DBD plasma device by using screen-printing technology to study immuno-stimulatory effects in metastatic cancers that are responsible for cancer relapse mediated by mesenchymal shift after therapies. Using microarray analysis, they postulated that plasma could stimulate and differentiate M1 pro-inflammatory macrophages which favor antitumorigenic immune responses in metastatic cancers via and cancer stem cell maintenance and metastasis acquisition. Figure 12 shows that the use of plasma technology could provide a breakthrough in modifying the pro-tumor inflammatory microenvironment by affecting resistant immunosuppressive tumor cells generally accompanying cancer relapse [16]. Nevertheless, this immune-modulation research area is quite young and reports on immune infiltrate in plasma-exposed animal tumor models are rare. Bearing in mind the power of an adaptive immune system, recently few antibody-based immunotherapeutic approaches have been the major innovation in the twenty-first century [152]. This concept is based on the fact that damage-associated molecular patterns are released from the dying tumor cells that help in the induction of ICD and tumor-specific cytotoxic T cells [153, 154]. Similar to the progress of other physical therapies for anticancer approaches for example ionizing radiation [155], electrochemotherapy [156], and photodynamic therapy [157], revolutionary outcomes in the area of immuno-oncology are probable stimulus research in future plasma medicine in near future.

**Fig. 13:**
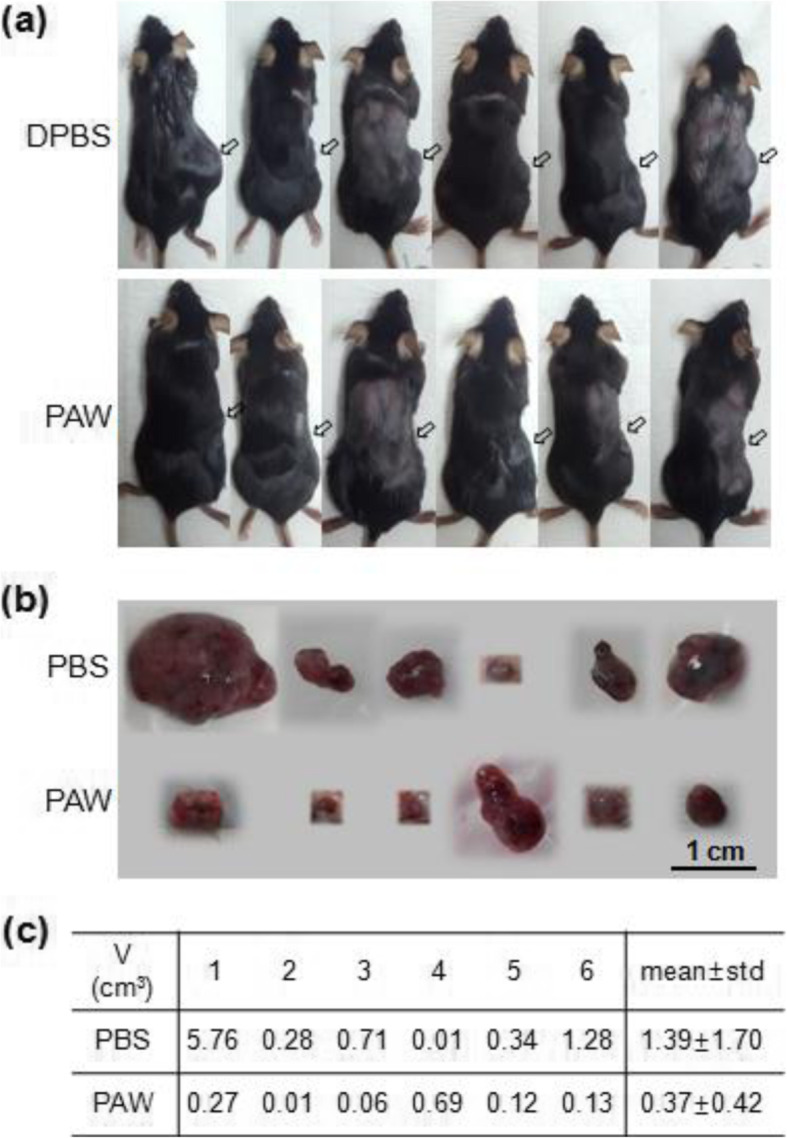
The anticancer effects of NO-PAW in vivo. **a** Pictures of 12 mice with melanoma syngeneic mice after 12 days of administration of DPBS and PAW. Arrows mark the positions of the tumor. **b** Pictures of tumor tissue from each mice shown in Fig. 11a. **c** Table showing the volumes of tumor tissues from the two groups [158]

In another study, a microwave jet plasma system produces nitric oxide called nitric oxide-plasma-activated water (NO-PAW). A previous report [158] explored the effects of NO-PAW on cancer cell lines, in comparison with other plasma devices. A further study [159] on the role of microwave plasma-generated NO-PAW on macrophage polarization was investigated. NO-PAW upregulates M1-type macrophage activation and downregulates the characteristics of M2-type macrophage at the transcriptional level. The anticancer potential of NO-PAW was also investigated in a syngeneic mouse model, as shown in Fig. 13. The results showed that NO-PAW has the potential to convert the fate of macrophages, suggesting this treatment as a supportive method for controlling the function of macrophages under the tumor microenvironment.

#### Plasma-activated medium therapy for cancer cell killing

To date, the biggest challenge for the plasma community is the treatment of in-depth cancers. In general, direct treatment of plasma can induce strong cell death in tissues by influencing the active species to the cells. Yet, only localized treatments are possible. Therefore, the question of “depth of penetration” requires our consideration. In this regard, researchers developed a process of delivering “plasma active species” to deep organ tissues could be through plasma-activated solutions. Indirect treatment of plasma is a method where reactive species are produced in a solution by treating plasma to a physiological buffer or medium. While this method causes less cell death than the direct plasma treatment, it has the least toxic effects on normal cells. Also, plasma-generated reactive species could be stored and can be treated in huge areas with easy handling. Studies have proven that plasma-activated medium (PAM) generated by indirect treatment can be a good candidate for biomedical applications such as cancer therapy [160]. Based on this approach, Utsumi et al. [161] also investigated that injection of plasma-activated medium (PAM) suppresses the tumor size plasma-activated at the tumor site. This result suggests that plasma application not only directly affects the cells but also indirectly prompts physiological moderators that may affect cells by creating reactive species around cells. Now, PAM could be proposed as a type of cancer chemotherapy. The plasma produced active species or plasma itself interacts with solutions in the liquid phase to generate H_2_O_2_, nitrates, and so on. Hence, PAM may be considered as a cocktail of several products irradiated with plasma and induces reactive species in cells. A similar study was performed by Ara et al. using a 2.45-GHz microwave-excited atmospheric pressure plasma jet device. Through measurement of various reactive species in medium, they concluded that among numerous reactive species produced by PAM, a high amount of H_2_O_2_ plays a vital role in inducing anticancer effects by PAM which was detected by high-level intracellular oxidative stress and was accompanied by apoptosis induction in lung cancer cells [162].

In the above study, PAM has been shown its potential to kill both chemo-resistant ovarian cancer cells and their parent cell lines [161]. Our group researchers also tried to highlight the effect of PAM on melanoma by detecting the changes at the molecular and biochemical level using the blood serum of mice. They established a new perception to understanding the mechanism of PAM for treating melanoma. Their result showed that the accumulation of the reactive species consisting in the PAM makes it a better treatment option against cancer cells as compared to direct plasma treatment as visualized by a decrease in protein and lipid levels in melanoma tissue. The specific melanoma LDH and L-DOPA prognostic markers were found to decrease, present in the blood serum and melanoma samples [163]. Liedtke et al. also observed that repeated intraperitoneal treatment of PAM suppresses the tumor growth in vivo as determined by magnetic resonance imaging, leading to reduced tumor mass and improved median survival (61 vs 52 days). Tumor nodes treated by PAM confirmed apoptotic areas with majorly inhibited cell proliferation. Also, the concentration of various cytokines in blood plasma, as well as the distribution of hematological parameters, was not changed by PAM treatment. They claimed that having minimal side effects of plasma-treated solutions, making them a feasible therapeutic candidate for advanced tumors [164]. As tumor relapse is a major hurdle in anticancer treatments, Elena et al. tried to use the plasma-activated physiological buffer solution (PBS and 0.9% NaCl) to mimic the clinical application of plasma-treated liquids. Using a 3D cellular model system, they suggested that the efficiency of PBS exposed to plasma was higher as it can penetrate deeper and faster as compared to NaCl exposed to the plasma, suggesting the use of plasma-exposed PBS for the treatment of in vivo tumors. Depending on the tumor site, these solutions can be injected directly into tumors (as in subcutaneous xenografts), or intraperitoneally when treating intraperitoneal malignancies (such as in the ovarian tumor model) [165]. Because treatment of solutions by plasma, due to their content of reactive species, may respond to ovarian cancer, which could be used in combination with other existing therapies, in this regard, Italy group of researchers developed a novel strategy that lies in the usage of a renowned clinically suitable fluid, i.e., Ringer’s lactate (RL). They stated that plasma-activated RL has a degree of selectivity towards cancer cells as compared to fibroblasts; however, further research needs to verify the exact mechanism underlying such favored anticancer activity [166].

Moreover, Tanaka et al. found that plasma-treated RL has shown good efficiency to exhibit an antitumor effect on glioblastomas in vivo and in vitro [167]. Their extended study demonstrates that both plasma-treated RL and PAM displayed antitumor effects on brain cancer cells. Nonetheless, their sensitivities were different as brain cancer cells shown more sensitivity to plasma-treated RL than PAM. The sensitivities of cells to plasma-activated liquids are thought to be determined by various factors which negatively and positively exhibit anticancer effects. Based on their findings they concluded that both the treated solutions induce anticancer effects on glioblastoma cells by distinctive mechanisms [168]. Moreover, to clear their understanding between PAM and plasma-activated RL solutions observed effects, they performed another study and witnessed that plasma-activated RL solution exhibited changes in intracellular metabolites of glioblastoma cells, as detected by the ratio of oxidative/reductive glutathione concentrations. In the metabolomic profiles of plasma-treated RL solution-treated cells, the generation of acetyl-CoA enhanced lipid metabolism. Based on these findings, they concluded that plasma-activated thus induces regulated death of U251SP glioblastoma cells in more innate microenvironments than PAM [169]. A similar group of researchers also tested the effects of plasma-treated RL solution on supported lipid bilayers (SLBs) as a cell membrane model. High-speed atomic force microscopy revealed that there were alterations in the morphological dynamics of SLBs when treated with plasma-treated RL solution, leading to a 20-fold decrease in SLB islands compared to without plasma-treated RL solution. These observed effects were basically due to the removal of lipid molecules from the edges of SLBs and their shrinkage [170]. It is also seen that PAM treatment can promote increased autophagic cell death in endometrial cancer cells in a concentration-dependent manner through inactivated mTOR pathway, which is critical for cancer cell viability.

However, future studies are required to investigate whether PAM treatment can reduce the recurrence rate of peritoneal metastasis using xenograft animal models [84]. More studies using PAM applications on residual cancer initiation cells along with EMT effect for anticancer approaches have been thoroughly discussed by Kaushik et al. [171]*.*

### Nonthermal plasma for wound healing application

Cold plasma can support wound healing by its antimicrobial effects, by activation and migration of skin cells such as fibroblast, keratinocytes, and melanocytes, and by activation of biological receptors (such as integrins) and by stimulation or regulation of immune cells [9]. Cold plasma active constituents can be delivered to target tissue, organ, and cells without making thermal damage. Due to this property of plasma, it has also gained attention for dermatological application. Plasma constituents, mainly ROS and RNS, can activate or regulate various complex biological reactions and pathways in a dose-dependent manner [9].

The human skin is the outer covering of the body and is the largest organ of the integumentary system. The skin is the best organ for direct or indirect plasma treatment in the human body. Plasma can provide an immediate therapeutic effect on skin lesions and can enhance the penetration of transdermal drugs or substances, thus avoiding the systemic drug’s toxic side effects [172].

**Fig. 14:**
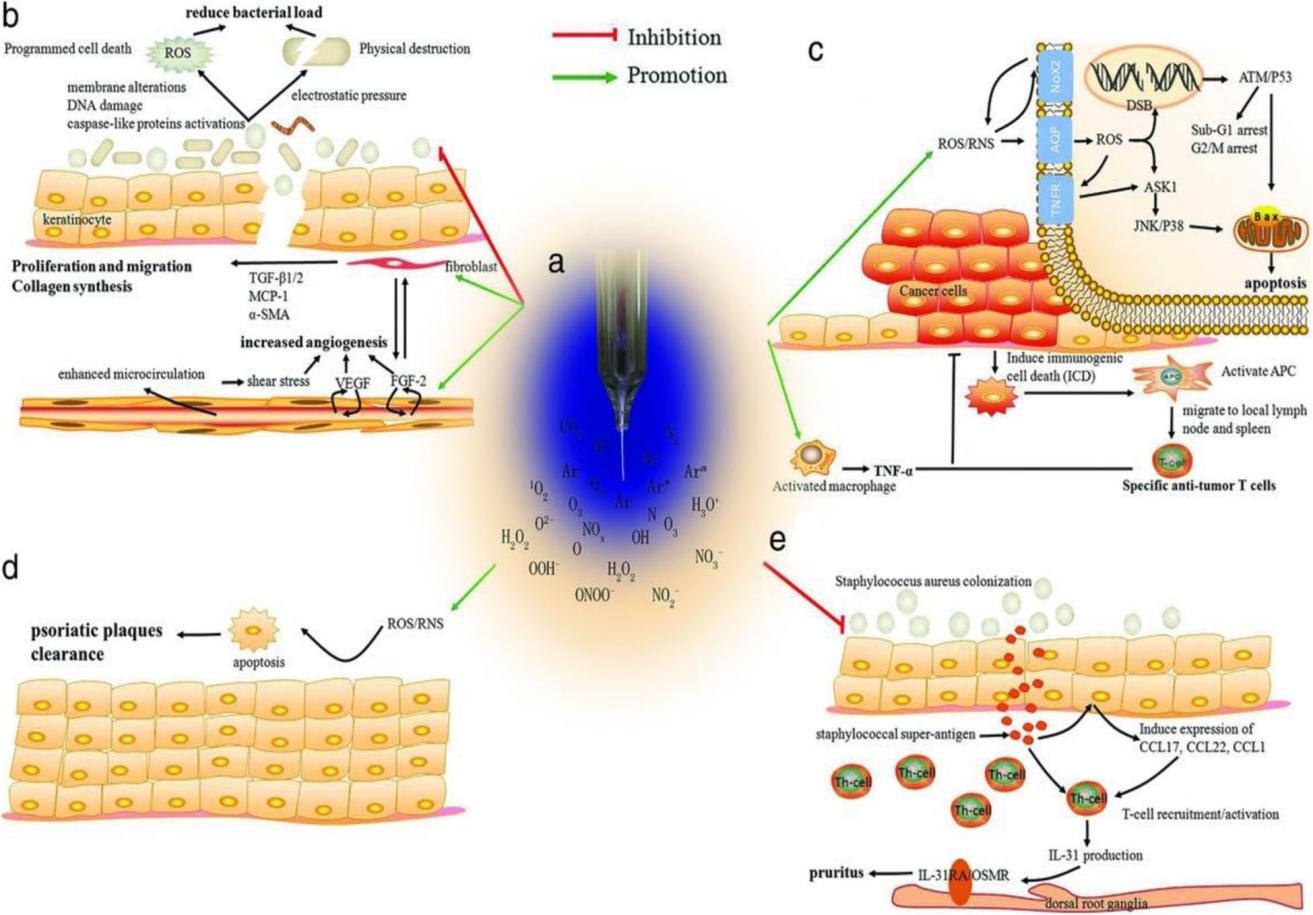
Summary of cold plasma-based wound healing therapeutic mechanism. **a** Nonthermal plasma and its constituents or active species. **b** Wound healing: plasma-induced reactive species inactivates pathogenic bacteria, activates skin cells, and improves angiogenesis and microcirculation. **c** Skin cancer: nonthermal plasma induces cell death, lethal nucleic acid damage, and cell cycle arrest in cancer cells. Also, plasma-based immunomodulation recruits systemic immune action. **d** Plasma-based psoriasis treatment. **e** Plasma-based atopic dermatitis and pruritus treatment. Copyright obtained from [172] 2017 John Wiley and Sons

Recently, small plasma devices (micron size) have been developed to be used inside the human body in a similar fashion to plasma-activated liquid or media. Small size plasma devices exhibit enhanced infiltrating capability and can be implanted into lesions or body cavities. In a recent study, a parallel plate dielectric barrier discharge and a capillary-guided corona discharge plasma device were used to treat chronic wounds [21]. Multidrug-resistant microorganisms are mainly responsible for recalcitrant to chronic wound healing. Methicillin-resistant *Staphylococcus aureus* and *Pseudomonas aeruginosa* are two main microorganisms in infected and clinically non-infected lesions. This report suggests that cold plasma can efficiently inactivate three different isolates of MRSA and four isolates of *P. aeruginosa* related to chronic wounds. Another study to investigate the disinfection rate using FE-DBD cold plasma [173] on planktonic and biofilm forms of *Escherichia coli*, *S. aureus*, and clinical isolates of multidrug-resistant MRSA-95 (clinical isolate), MRSA-USA300, and MRSA-USA400 reported that the bacteria colony counts were reduced significantly in less than 60 s and completely disinfected in ≤120 s. The energy dissipated in this FE-DBD plasma was estimated to be around 7.8 J/cm^2^. Another study shows complete inactivation of *Escherichia coli* using homogeneous helium/air DBD discharge with 2–20 min exposure time. Many other researchers also support plasma inactivation of bacterial cells and discuss the mechanisms of plasma antimicrobial effects and proliferation and regulation of cells in the wound area and plasma components’ relevance in the wound healing process [174–179] (Fig. 14).

Blood coagulation is also an important process in wound healing. Recent in vitro and in vivo studies also suggest the potential role of plasma to control bleeding lesions with relevance to the gas used in plasma devices. It is indicated that that nitrogen and carbon dioxide gas cold plasma jets have the potential to be used for hemostasis in various surgical areas [180].

Treatment of burn wounds is another serious issue worldwide. More than 6 million patients seek medical attention due to burning cases every year. Recently, nonthermal plasma is also investigated for treating burn wounds. A study assessed the application of cold plasma jet on the brass comb burn wound model in rats [181]. This study reported that plasma decreased the burn injury progression in the unburned interspaces in a rat comb burn model by reducing necrosis and improving the healing process.

Also, in another study, microplasma jet array device fabricated from a transparent polymer is used to investigate burn wounds [182]. The microplasma jet device can efficiently kill fungus and bacterial in both in vitro and in vivo conditions and showed a significant wound healing effect by affecting immune factors such as interleukins and regulation of inflammatory genes. Microplasma jet arrays can have advantages over single-jet devices such as superior packing density, characteristic scalability for bigger treatment areas, unparalleled material flexibility in a plasma jet device, and the selective production of biologically relevant species at higher plasma densities. Not only burn wounds, pressure ulcer treatment is also another problem in the field of dermatology due to modern lifestyle and underlying long-term sickness. A recent study reported the application of nonthermal atmospheric pressure plasma for the treatment of pressure ulcer wounds. The study related to non-invasive nonthermal plasma treatment to pressure ulcer treatment showed that plasma accelerated the healing process of pressure sores and can be safely applied to the human body [183]. This study showed plasma for a mechanical and histological parameter of treated tissue can be tuned by using optimal power, plasma temperature, controlling the input voltage and the distance from the plasma device surface. It is also important to know the proper treatment time for internal organ wounds. A study on an internal wound on the Balb/c mice is conducted to investigate to check helium jet plasma treatment or exposure time as the time required for blood coagulation and wound healing after treatment [184]. This report shows 30 s argon plasma jet treatment can induce better wound healing in the cut liver in mice models. It is reported that 15% of the patient with diabetes mellitus worldwide agonize from diabetic ulcers.

Recently, nonthermal plasma treatment is also investigated as a novel therapy for diabetic ulcers. A study compared the healing rate in diabetic and non-diabetic wounds in mice models [185]. Investigation showed plasma reduced wounds in both diabetic and non-diabetic wounds. However, plasma treatment showed a low healing rate in diabetic wounds compared with non-diabetic animals. Histological analyses also revealed the induction of an epidermis layer formation, TGF-β1 cytokine signaling factor, neovascularization, and cell proliferation after plasma treatment. Also, to support the safety of plasma devices for wound treatment a study is conducted to evaluate acute toxicity in porcine skin tissues [186]. This study reported that FE-DBD cold plasma can safely be applied to intact skin for up to 2 min at a plasma power of 0.17 W/cm^2^ (20.4 J/cm^2^) without causing any macroscopic and microscopic tissue damage. At wounded skin, plasma treatment induced blood coagulation properties to form an early clot, which possibly shields against any tissue damage for up to at least 15 min plasma exposure. In this study, a pilot scale can be recognized to define thresholds for further investigations into dermatological applications of cold plasma. A further report on acute wound healing by plasma treatment highlighted the importance of cold plasma applications for wound therapy [187]. Among the main advantages of plasma treatment showed no side effects and associated with activation of pro-inflammatory signaling pathways. Another investigation supported activation of STAT3 signaling without apoptosis by plasma to promote myogenic differentiation by controlling the expression of MyoD, myogenin, and MyHC [188]. Nonthermal plasma treatment initiates nonlethal oxidative cell stimulation that promotes myogenic differentiation. Nonthermal N2/Ar microplasma exposure also showed no heat-associated damaging effect in wound tissue [189]. Cold N2/Ar microplasma exposure also showed enhanced wound healing in the wound region in animal models. Plasma treatment specific organ toxicity was also investigated in Sprague-Dawley rat models as a part of a safety study [190]. This report also suggested that short-term, low-dose plasma treatment to wounds accelerated the wound healing process in rats without having any indication of vital organ toxicity. These data support an alternative and safe therapeutic use of plasma devices with a low operating temperature and adjustable plasma parameters for wound management in clinical settings.

Not only direct wound treatments, nonthermal plasma also been used to treat wound dressing recently. Plasma-treated alginate wound dressing has shown strong antiseptic and antibacterial properties against resistant microbes [191]. In this study, anti-biofilm efficacy of the plasma-treated alginate gel and cytotoxic effect on endothelial cells by the treated gel was investigated. The treated gel was tested against six microbes and the thickness of dressing and distance between the plasma surface and the gel surface were kept constant. Cold plasma 1 and 3 min exposed wound dressing gel inactivated all the six microbes included in the study at counts of 10^9^ CFU/mL of the microorganisms without any toxicity to endothelial cells including migration and proliferation rate of these normal cells. Interestingly, this plasma-treated dressing material retained its antimicrobial effects for more than a month. While the enhancement of wound healing by plasma treatment has been described in several reports, the molecular influences on human tissues are also an important parameter to characterize wound healing devices. In another important study, human S9 bronchial epithelial cells were treated with nonthermal atmospheric pressure jet plasma that was previously shown to improve the wound healing process [192]. Several new parameters and techniques were included in this study to investigate short-term (up to 1 h) and long-term (24–72 h) effect by plasma using proteome analyses on human epithelial cells. K-means cluster analysis and time-related analysis of fold-change factors. Short-term results indicated the induction of prevention of apoptosis, Nrf2-mediated oxidative, PPAR-alpha/RXR activation, and endoplasmic reticulum stress response, as well as the production of peroxisomes. These effects are important to overcome cellular oxidative stress and to maintain the integrity and specifically microtubule dynamics. These indicators can be further used for risk assessment and quality management of wound healing application of cold plasma in clinical settings. These findings suggest a possible mechanism that either motivates cells into cell death or mounts adaptive responses letting them survive after nonthermal plasma treatment. These observations suggest that plasma can turn a chronic wound into a less dangerous acute wound with a better curing possibility. Based on all previous reports, the basic mechanisms of plasma effects on wound healing are mainly via ROS and RNS which were explained in five steps: (i) speeding up re-epithelialization and wound closure, (ii) regulation of inflammation via protective mechanisms (such as ß-defensins and NRF2 signaling) and immune cells recruitment in the wound area, (iii) fibroblasts activation to regulate actin cytoskeleton and to activate the collagen synthesis, (iv) activation of growth factors in skin cells and cytokines as immune regulation relevant to wound healing process, and (v) stimulate neovascularization process [192–194].

A clinical study on plasma treatment-based wound healing process also showed promising results. A study indicated plasma can accelerate the wound healing process even after oral surgery related to gingival treatment [195] and skin graft surgery in patients [196]. The use of a portable DBD plasma (PlasmaDerm® VU-2010) is also investigated to alleviate chronic venous leg ulcers in a monocentric, two-armed, open, prospective, randomized, and controlled trial [197]. Further prospective clinical studies using a different plasma device primarily aimed at decreasing the bacterial load in chronic wounds and accelerating wound healing process reported promising findings [198–207]. More clinical studies are needed to determine the most appropriate treatment options for patients.

The recent results presented in this review show that cold plasma could be effective in the inactivation-resistant microbe and fast-tracked wound healing process (Table 1). Also, cold plasma treatment of acute or chronic wounds has many advantages over more present conventional procedures used in clinics. Altogether, plasma treatment holds vast application and development prospects. Technical standards for the plasma devices to allow a consistent treatment of specific diseases are strongly needed. This can also be the foundation for the evaluation of the result of biological testing and clinical trials conducted in hospitals. In the future, plasma treatment wound healing efficacy must be demonstrated in standardized therapeutic strategies, randomized and larger clinical trials with confirmation of long-term biological safety.

**Table 1:** Overview clinical trials for plasma wound healing

Title	Number of subjects	Conclusion	Wound	Reference
**A first randomized clinical trial** to decrease bacterial load using cold atmospheric argon plasma on chronic wounds	36 patients	Highly significant reduction in bacterial load	Chronic	[200]
Successful use of 2 min cold atmospheric **argon plasma in chronic wound**s: results of a randomized controlled trial	24 patients	Highly significant reduction in bacterial load	Chronic	[199]
Cold atmospheric argon plasma treatment for **chronic wound** healing	70 patients	Wound healing may be accelerated by plasma, particularly for chronic venous ulcers	Chronic	[207]
The healing effect of **plasma in pressure ulcer**: a randomized controlled trial	50 patients	The plasma-treated group had significantly better PUSH (pressure ulcer scale for healing) scores and exudate amount	Chronic	[201]
Clinical use of argon plasma in **chronic leg ulcers**: a pilot study	16 patients	Immediate antimicrobial comparable to octenidine without signs of cytotoxicity	Chronic	[202]
Combined antibacterial effects of plasma and a modern conventional liquid antiseptic on chronic wound treatment	34 patients	The combined use of CAP and conventional antiseptics represent the most efficient strategy for the treatment of chronic wounds	Chronic	[203]
Alleviation of chronic venous leg ulcers by dielectric barrier discharge plasma	14 patients	PlasmaDerm® VU-2010 device is safe and effective in patients with chronic venous leg ulcers	Chronic	[197]
A randomized placebo-controlled human pilot study of argon plasma on skin graft donor sites	40 patients	Donor site wound areas treated with plasma showed significantly improved healing compared with placebo-treated areas	Acute	[196]
Experimental recovery of CO_2_-laser skin lesions by plasma stimulation	5 experimental case reports	Plasma stimulation of laser skin lesion recovery looks promising	Acute	[206]
Scar formation of laser skin lesions after plasma treatment: a clinical long-term observation	20 laser lesions in 5 individuals	Plasma treatment seems to support the inflammation needed for tissue regeneration	Acute	[205]
Laser scanning microscopy to assess the augmentation of tissue repair by an exposition of wounds to plasma	6 subjects with vacuum generated wounds	Plasma led to a significantly more rapid area decline in comparison to no treatment, octenidine treatment, and a combination	Acute	[204]

### Plasma antibacterial effects

#### Deactivation of bacteria from food products using plasma

In developing modern and busy lifestyle, most of the people, especially in the developed countries, rely mainly on packed and stored food packages for survival. In this aspect, food preservation is a constant fight against pathogenic microorganisms and induced contamination in foods. Therefore, the improvement in food quality and safety without compromising the nutritional values has led to a growing interest in low-temperature innovative processes for food preservation worldwide. In recent times, nonthermal plasma is a unique technique that can inactivate microbial spores on surfaces during the processing of solid food as well as of liquid products [47]. Also, it is an excellent alternative method to use for the deactivation of foodborne microbes without harming food appearance and quality [208]. This present approach gives an idea for the plasma application in bio-decontamination processes. Since disease outbreaks have been constantly enhanced from fresh produce, Igbal et al. developed a plasma-based method to deactivate the biofilm of *Aeromonas hydrophila*, and food-borne pathogen present on lettuce. They stated that only 5 min of plasma treatment effectively reduced the growth of *A. hydrophila* biofilms on lettuce at less than 15 °C. However, these biofilms were resistant to plasma at higher temperatures (≥ 15 °C). These studies highlight the influence of temperature on storing produce to inhibit pathogen formation on lettuce treated by plasma [48]. A similar effect was also observed on antibacterial activity against *Escherichia coli*, *Salmonella enterica Typhimurium*, and *Listeria monocytogenes* injected on fresh strawberries and cherry tomatoes, by plasma. They stated that approximately 300-s plasma treatment shows almost undetectable levels of these microbes on tomatoes’ and cherries’ surfaces inside a sealed package [49]. This approach has the prospective to offer both increased shelf-life and microbiological food safety. In another study, the decontamination ability of resistive barrier discharge (RBD) prototype gas plasma was analyzed on shell eggs inoculated with two bacterial strains, named *Salmonella enteritidis* and *Salmonella Typhimurium* (5.5–6.5 Log CFU/egg shell). They exposed these samples in the plasma after-glow chamber to decrease the risk of egg quality alteration where the temperature was near to the room temperature. Maximum bacterial load declines of about 2.5 and 4.5 Log CFU/eggshell were attained for *S. enteritidis* after 90 min of exposure without harming egg quality traits. This method showed a feasibility for the poultry industries that usually stock eggs for a longer period of time. Additional benefit of this plasma set up is the use of atmosphere itself as working gas and the low temperature (∼ 25 °C), which makes it considerable as a cost-effective, and interesting equipment for the industry [209].

The above studies provided evidence that plasma has the potential to inactivate microbes mainly present on food surfaces; nevertheless, less consideration has been paid to the influence of plasma to deactivate microbes in liquid foodstuffs. By taking this into the consideration, Gurol et al. used plasma technology for the elimination of bacteria from daily products. Interestingly, they observed the successful decontamination capability of plasma from *E. coli* in milk samples regardless of the fat content of the milk, after 20 min of treatment. Even after 1 week of examination in full-fat milk samples, no live cells were noticed and continued over a longer storage period without any milk color change. These studies provide hope to use plasma devices to decontaminate dairy products with an extension of the product’s shelf life while maintaining its nutritional properties [50]. An enhanced ability to inactivate *Citrobacter freundii*, generally present in apple juice, was also observed by argon/O_2_ plasma application after 480 s of exposure. They stated that direct contact among bacterial cells and plasma is not particularly required for achieving complete deactivation. The plasma-induced reactive compounds in the liquid phase, such as H_2_O_2_, are likely responsible for these antimicrobial activities by inducing cellular permeabilization and RNA damage [51]. These preliminary studies facilitate further investigation on the stability of quality-determining factors to validate their safety concern for human consumption.

#### Efficient bacterial killing from various surfaces using plasma

**Fig. 15:**
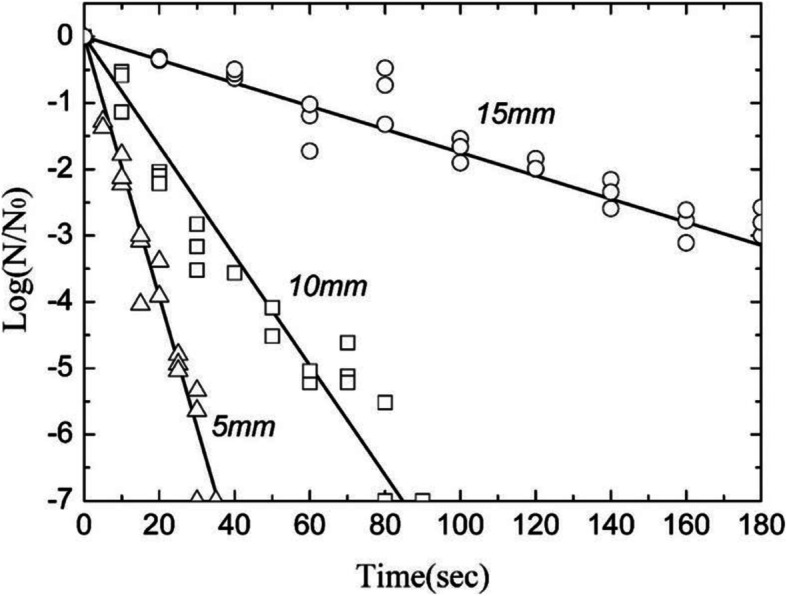
Plots of the death curves Log(*N*/*N*_0_) versus the exposure distance. Control colony number *N*_0_ ≈ 3.3 × 10^6^ cfu/ml. Input power = 130 W, Ar gas flow = 10 lpm, O_2_ gas flow = 25 ccm (0.25 vol.%), and exposure distance = 0.5 cm (triangle), 1 cm (square), and 1.5 cm (circle) [211]

**Fig. 16:**
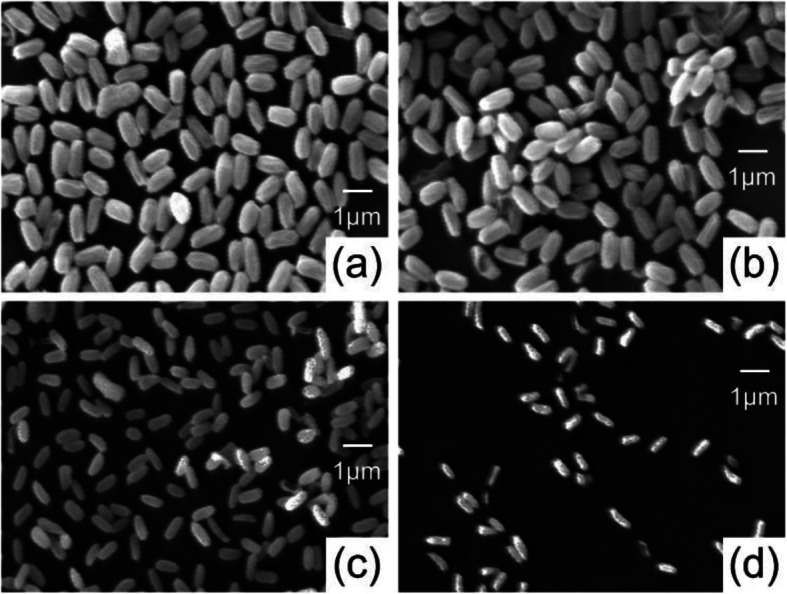
Scanning electron micrographs (× 10,000) of *B. atrophaeus* spores: **a** untreated, **b** treated with heating gas (90 °C, 30 s), **c** treated with Ar/O_2_ plasma jet (85 °C, 20 s), and **d** treated with Ar/O_2_ plasma jet (85 °C, 30 s). The atmospheric pressure Ar/O_2_ plasma jet was operated at 130 W, 0.25 vol.% O_2_, and 0.5 cm of the exposure distance. [211]

One of the key issues in the plasma jet is the sterilization of anthrax spores, which are the deadliest microbes for biological warfare agents. In this context, a high-power rf plasma source was motivated to be developed. To enhance discharge characteristics, a stepped electrode concept was introduced [210]. The stepped electrode reactor makes it easy to operate Ar/O_2_ glow discharge, providing a stable, uniform, and broad plasma jet at atmospheric pressure. Eventually, a cylindrical plasma jet operated by rf frequency was developed [211–213] for sterilization of anthrax spores. Argon plasma jets penetrate deep into ambient air and create a path for oxygen radicals to sterilize microbes. A sterilization experiment with bacterial endospores indicates that an argon-oxygen plasma jet very effectively kills endospores of *Bacillus atrophaeus* (ATCC 9372), thereby demonstrating its capability to clean surfaces and its usefulness for reinstating contaminated equipment as free from toxic biological warfare agents. The decimal reduction time (*D* values) of the Ar/O_2_ plasma jet at an exposure distance of 0.5–1.5 cm ranges from 5 to 57 s, as shown in Fig. 15. An actinometric comparison of the sterilization data shows that atomic oxygen radicals play a significant role in plasma sterilization. When observed under a scanning electron microscope, the average size of the spores appears to be greatly reduced due to chemical reactions with the oxygen radicals in Fig. 16.

As biofilm formation, a bacterial ability to protect itself in a polysaccharide and protein-containing matrix, is a major healthcare issue for hospital infections, Tripti et al. employed the plasma approach to preventing this issue in a safe and cost-affordable manner. Complete sterilization of *P. aeruginosa* biofilms can be achieved after plasma and chlorhexidine digluconate co-treatment. This combined treatment approach could be considered for complete sterilization of medical devices and biomaterials surfaces using safe doses of plasma and chlorhexidine digluconate [52]. Critically, these techniques could only provide sterilized substances up to the end of the process but not afterward due to the lack of pre-packaging items which is a necessity to ensure their disinfected state during shipping and storage prior to their use. To overcome this issue, Ben Belgacem et al. developed a plasma generator inside a sealed bag to evaluate its sterilization efficacy against *Staphylococcus aureus*, *Pseudomonas aeruginosa*, and *Bacillus subtilis* spores on their equipment (patent WO 2012/038669 A1) inside the sealed bag. Plasma treatment kills the abacterial strain within 120 min of exposure without any visible damage to the bags as preserving the bag integrity is a prerequisite to preserving the sterile state of the sterilized medical devices. This ground-breaking technique represents a successful attempt in the plasma sterilization applications for medical devices and preventing toxic risks for the workers [53]. As biofilm formation is a major cause to develop bacterial resistance that makes them hard to deactivate, in this regard, Paldrychová et al. used plasma alone or with a combination of antibiotics to eradicate the mature biofilm of *Pseudomonas aeruginosa* formed on titanium surfaces. This work suggests the use of a combination of plasma and antibiotics by improving the effects of those antibiotics, which extends their use in clinical practice [54]. Other teams of researchers have also shown that the bacterial adhesion and biofilm formation were dramatically suppressed on the plasma-treated titanium surfaces used for dental implants through causing bacterial cell wall oxidation which was more sensitive to gram-negative bacteria. These findings provide the importance of plasma not only for preventing infection before implantation but also for reducing bacterial contamination during the implant surgical process that usually leads to implantation failure [55]. *Propionibacterium acnes* (*P. acnes*) is an opportunistic gram-positive pathogen that becomes an important source of various surgical implant-associated infections including artificial joints, shunts, heart valves, and catheter infections. The inactivation ability of two different plasma jets, nonthermal annular plasma jet (NAPJ), and nonthermal soft plasma jet (NSPJ) for *P. acnes* in planktonic state and biofilm state was studied [214]. It was found that both plasma devices showed considerable inactivation potential in planktonic *P. acnes* and *P. acnes* biofilms. Especially, NSPJ showed a better inhibitory effect in a shorter exposer time than NAPJ which might be because of close exposure to plasma-generated reactive species. To mimic some in vivo conditions, Seo et al. attempted to treat plasma on artificial saliva, which indicates that plasma could be more applicable in a physiological oral environment with saliva rather than under certain experimental settings [56]. A similar approach was used to decontaminate surgical wounds where the surgical site infection rate is higher. Scientists used the plasma technique to reduce the degree of on-site infection. Plasma treatment appears safe for the treatment of epithelialized and granulating wound surfaces with decontamination of *Staphylococcus aureus*. More investigations on the plasma effect on such responses will indicate its clinical applicability as delivery device [57]. Although experimental work has focused on plasma application as a successful antiseptic for chronic and infected wounds via deactivating bacterial growth, the molecular mechanisms underlying these effects are less stated. In 2020, Persson et al. demonstrated that plasma can abolish the ability of M1 protein, a *Streptococcus pyogenes* virulence determinant that triggers inflammatory host responses through oxidation of Met81 and Trp128 placed at the sub-N-terminal region of M1 protein without any vital effect on the host immune system. These studies may open new research avenues for the further development approaches for the treatment of wound infections caused by *S. pyogenes* [215].

The use of DBD plasma to inactivate aerosolized *Bacillus subtilis* and *Pseudomonas fluorescens* considering indoor and outdoor bioaerosols. Investigational data confirmed that plasma exposure caused remarkable inactivation of environmental bacterial bioaerosols within milliseconds both indoors and outdoors. Plasma technology holds great potential in inactivating bioaerosols, particularly in environments that are contaminated accidentally by infectious agents. Such applications could also extend to air sterilization in hospitals and other public health facilities. These findings suggest that plasma could be a powerful tool for air decontamination technology [216]. Recently, our group developed a commercial compact plasma sterilizer through DBD setup that can disinfect the air by eradiating bacterial strains including viruses. This new plasma sterilizer displays excellent sterilization properties, without causing environmental contamination problems as induced by conventional chemical materials. It is worth mentioning that this plasma sterilizer can also remove air dust particles (air purification) as well as prevent secondary infection during patient transport except for the removal of microorganisms [217]. Additionally, another group of researchers defined that plasma inactivates bacterial cells by cell surface damage, which further leads to the loss of membrane integrity, leakage of intracellular components such as nucleic acid, and protein in multidrug-resistant bacteria named *Staphylococcus aureus* (MRSA), *Pseudomonas aeruginosa*, and *Candida albicans*. Mahmoud et al. also demonstrated that drug-resistant *Bacillus cereus*, *Staphylococcus aureus* (MRSA), *Escherichia coli*, and *Pseudomonas aeruginosa* were completely deactivated within 2 min of jet plasma exposure through cell membrane damage, and permeability perturbation as a primary mechanism. This study verifies that plasma promotes a non-selective, multiple-target mechanism of action, to overcome microbial resistance [218]. Plasma treatment of multidrug-resistant bacteria was studied [119], where two types of plasma systems were investigated: nanosecond pulsed plasma (NPP) as liquid discharge plasma and an Argon gas-feeding dielectric barrier discharge (Ar-DBD) as a form of surface plasma. It was observed that both plasma sources can inactivate both the wild-type and multidrug-resistant bacteria to a good extent. Moreover, it was observed a change in the surface morphology, gene expression, and β-lactamase activity.

Consistent with the above notion, these findings suggest the use of plasmas for disinfection of multidrug-resistant microorganisms in the environment. Since plasma treatment is superficial, the physical barrier of the cell is a critical factor of plasma-induced sterilization efficacy [219]. An enhanced disinfection effect was also observed against *Bacillus subtilis* and *Escherichia coli* when DBD plasma was applied using different working gases, i.e., Ar diluted with 25% O_2_ [220]. On the other hand, Theinkom et al. showed that membrane damage does not seem to be the primary mechanism of action for plasma against *Enterococcus faecalis* [221]. Plasma application was also used to manage hospital wastes which can have adverse effects on human infection and induced bacterial resistance. Maryam developed a DBD plasma-based method for effective bacterial deactivation within a short exposure time via the destruction of *Pseudomonas aeruginosa* and *Staphylococcus aureus* internal cellular structures. This strategy provides a simple and eco-friendly procedure in which hazardous wastes can be cleansed without harming the operator to make it an excellent option for hospital waste sterilization [222].

**Fig. 17:**
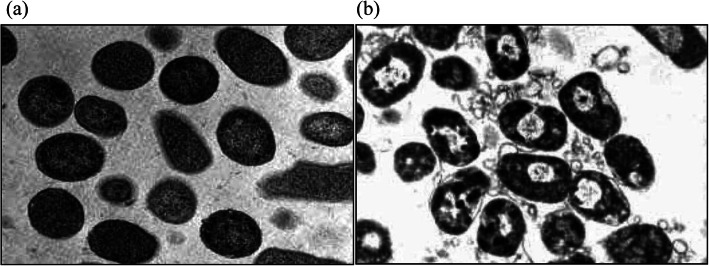
Transmission electron microscopy of *E. coli* (× 4400): **a** control cells and **b** cells after 2 min of plasma treatment at 75 W [224]

A comprehensive understanding of the influence of reactive oxygen species on microbe sterilization was also presented by our group [223]. A sterilization experiment with bacterial endospores reveals that an argon-oxygen plasma jet very effectively kills endospores of *Bacillus atrophaeus* (ATCC 9372), thereby indicating that oxygen radicals are the key element of sterilization. Figure 17 shows a dramatic image of how microbes are effectively destroyed by plasma jet, showing spilling of micro chromosome from cells. Ozone in acidic water also kills endospores of *B. atrophaeus* very effectively, demonstrating the capability of cleaning a large surface area contaminated by toxic biological agents. However, advanced cells such as fertilized eggs were not greatly influenced by the acidic ozone water. Also, both human and canine cells after treatment with the acidic ozone water prospered without showing signs of stress due to ozone in acidic water. This study suggested that antioxidant enzymes such as superoxide dismutase can be developed in the advanced cells to protect themselves from attacks by reactive oxygen species. Meanwhile, the advanced cells utilize oxygen by certain enzymes, proliferating life on earth.

The effects of air and N_2_ plasma jets on *Escherichia coli* inside four different physiological solutions and deionized water were reported [225]. The feeding gases were found to influence the plasma compositions, the interactions with the liquids, and the subsequent bactericidal effects depending on the aqueous solutions to which they are exposed. Air plasma enhances the bactericidal effects of deionized water and saline by acidification. On the other hand, N_2_ plasma induced higher toxicity in phosphate-buffered saline by generating more electrons.

Plasma-treated water (PTW) for inactivation of microorganisms was investigated [78], studying the role of reactive nitrogen species (RNS) in the deactivation of bacteria for the development of a plasma system with increased sterilization efficiency. To increase the concentration of reactive oxygen and nitrogen species (RONS) in solution, vapor systems were used (DI water/HNO_3_ at different wt%) combined with plasma using N_2_ as working gas. Interestingly, more deactivation of *E. coli* with PTW created by N_2_ + 0.5 wt% HNO_3_ vapor plasma was observed as compared to PTW generated by the other plasma systems.

Mycobacterial cell walls comprise thick and diverse lipids and glycolipids that act as a permeability barrier to antibiotics or other chemical agents. The use of OH• radicals from a nonthermal plasma jet (NTPJ) for the inactivation of mycobacteria in an aqueous solution was adopted as a novel approach [226]. The addition of water vapor in a nitrogen plasma jet generated OH• radicals, which converted to hydrogen peroxide (H_2_O_2_) that inactivated non-pathogenic *Mycobacterium smegmatis* and pathogenic *Mycobacterium tuberculosis H37Rv*. Morphological changes of *M. smegmatis* and *M. tuberculosis H37Rv* were observed after 5 min treatment with N_2_ + H_2_O plasma, but not of the pre-incubated sample with catalase. This finding indicated that the bactericidal efficacy of NTPJ is related to the toxicity of OH• and H_2_O_2_ radicals in cells. Therefore, this study suggested that NTPJ treatment may effectively control pulmonary infections caused by *M. tuberculosis* and nontuberculous mycobacteria (NTM) such as *M. avium* or *M. abscessus* in water.

Although plasma technology has shown the excellent possibility to compete with existing methods, optimize the plasma ability for individual application is necessary. Plasma technology is showed as a powerful tool for disinfecting food surfaces, along with its packaging and storage. Another aspect of the future plasma technology is the possibility of pairing it with other decontamination processes for many effective processes.

### Antiviral effect of nonthermal plasma

Pathogenic viruses are a very serious problem of the present time that can cause serious consequences related to health and the environment. Existing procedures for virus inactivation or disinfection have serious disadvantages such as contamination of the environment by forming chemical residues or by-products with carcinogenic effects and inconvenience to use. Plasma can effectively inactivate or kill microbes such as bacteria; however, the antiviral effect is not fully explored previously. Recently, plasma medicine researchers initiated studies related to antiviral effects by using various nonthermal plasma devices. One recent study showed promising antiviral results on bacteriophage T4, Φ174, and MS2 strains using Argon gas surface plasma mixed with 1% air and its activated water [227]**.** Both, direct treatment by plasma and plasma-activated water showed inactivation of bacteriophages in a dose-dependent manner. The mechanism based on protein and DNA analysis showed that plasma damages nucleic acid and protein markers and cause aggregation of viruses. It is also noticed that singlet oxygen is a very important species in the process of virus killing. In another study, plasma-activated solutions are used to inactivate Newcastle Disease Virus (NDV) which is an infectious viral disease of domestic animal and poultry industries [228]. It is showed that reactive oxygen and nitrogen species play important role in NDV virus inactivation. RONS such as short-lived OH• and NO˙ and stable H_2_O_2_ damages or degrades nucleic acids and viral proteins and alters the morphology of the virus, consequently inducing virus inactivation. Therefore, the use of plasma-activated solutions which are green can be a promising alternative strategy in domestic animals and poultry industries. The inactivation of the airborne virus is also an important topic of study nowadays. The airborne virus can have a serious impact on the human respiratory syndrome. Recent results showed that cold plasma can effectively inactivate aerosolized MS2 phage in a dose-dependent manner [62]. A more than 2.3 log reduction of aerosolized MS2 virus by plasma generated at 30 kV with 170 standard liters per was achieved across the packed bed plasma reactor. Inactivation mechanisms of plasma-treated viruses must be explored in the future time. Airborne MS2 bacteriophages were treated using nonthermal plasma at 20, 24, and 28 W power levels and different gases such as He-O_2_ [2%, vol/vol], Ar-O_2_ [2%, vol/vol], ambient air [229]. Plasma treatment showed 95% inactivation (1.3 log reduction) at 28 W power and indicated a linear relationship between virus inactivation and plasma doses concerning power and exposure time. Another report in support of rapid inactivation of the airborne virus also showed that DBD plasma can induce in-flight inactivation of an airborne aerosolized porcine reproductive and respiratory syndrome (PPRS) virus [230]. The ∼ 3.5 log10 reductions in the virus titer are detected using the TCID50 method and viral genome damage is detected by reverse-transcription quantitative real-time polymerase chain reaction (RT-PCR) method. This is suggested that the inactivation effect is based on both short-lived (such as OH• and singlet oxygen) and long-lived (such as peoxynitrous) species with low pH. A study showed a method that evaluates DNA damage of the bacteriophage (*λ* phages) viruses in vivo treated with plasma in the air [231]. The assay includes DNA extraction from inactivated lambda bacteriophages and transfection to the host bacteria. Comparison between survival curves of the inactivated viruses and survival curves observed by the DNA transfection indicated DNA damage responsible for inactivation. There is another previous study on mechanism analysis of bacteriophage φX174 inactivation using atmospheric pressure DBD and corona discharge in dry and wet conditions [232]. It is analyzed that damage induced by both the coat protein and the DNA of bacteriophage φX174 after plasma treatment. It is also found that damage associated with coat protein is more prominent as compared with the damage on the DNA. Nowadays the development of various food processing and safety methods is also explored worldwide to meet the requirement of food material demand. Various food processing procedures are not very effective against viruses, such as human norovirus (NV). The in vitro virucidal activity on feline calicivirus (FCV), a surrogate of NV using cold plasma, is reported [233]. Factors affecting the treatment of FCV using plasma are treatment time, power, distance, feeding gas, and the virus suspension medium. This study showed the highest virucidal effect is by Ar plus 1% O_2_ gas among four plasma gas mixtures (Ar, Ar plus 1% O_2_, Ar plus 1% dry air, and Ar plus 0.27% water). Even plasma-activated buffered solutions are used in a study to inactivate feline calicivirus (FCV) [234]. The mechanism indicated that oxygen and air gas are predominant for inactivation as substantial pH reduction is induced in the buffer solution by the plasma. It is also important that singlet oxygen chemistry dominates with H_2_O_2_, O_3_ NOx to inactivated FCVs. Different chemical deactivation mechanisms can be realized with the same plasma device using various gas admixtures. Similar study on murine norona virus (MNV) and hepatitis A virus (HAV) in three types of fresh meats (pork shoulder, chicken breast, and beef loin) is also conducted [235]. The inactivation of MNV and HAV viral titers from three types of fresh meats elevated as the plasma dose increased. Noteworthy, this plasma treatment is not affecting meat quality up to 5 min exposure. Plasma treatment showed > 99% reduction (2 log_10_ PFU/mL) and > 90% reduction (1 log_10_ PFU/mL) of MNV-1 titer and HAV titer, respectively. Therefore, the plasma jet treatment can be a good alternative to cold treatment methods for high-fat food-borne viruses. A similar investigation on the cold plasma using oxygen gas reported inactivation of murine norovirus-1 (MNV-1) and hepatitis A virus (HAV) on stainless steel substrate. On such metal substrates, plasma exposure for 10–30 s reduced major food-borne MNV-1 and HAV to 0.65–3.89 and 0.77–2.02 log_10_ PFU/ml [236]. Another study related to the inactivation of tobacco plant-based tobacco mosaic virus (TMV) showed that plasma treatment can disrupt TMV particles and as well as nucleic acid to decrease their infection rate [237]. Inactivation of TMVs also depends on plasma treatment doses, can collapse the virus to parts, and can degrade its RNA moderate to high doses of plasma exposure. To investigate the exact mechanism of virus nanoparticle inactivation, a study was conducted on the respiratory syncytial virus (RSV), which has a diameter of 80–350 nm [224]. The production of various ROS, including neutral reactive species, is identified using a zero-dimensional kinetic universal model of the reaction scheme during gas plasma generation. This study showed that the inactivation of viruses is mainly due to neutral reactive species produced during plasma generation. Another study showed that nonthermal plasma also can target human immune deficiency virus (HIV-1) and virus-containing macrophage cells [238]. This study investigated the effects of nonthermal plasma on HIV replication in monocyte-derived macrophages (MDM). Treatment of MDM with plasma alleviates CD4 and CCR5 levels and can inhibit transcription, virus-cell fusion, and integration of subunits. Remarkably, plasma treatment to MDM cells reduced infectivity of virus particles and also indicating that the significant inhibitory activity can be attained up to the second cycle of infection. The nonthermal He gas plasma is also used to inhibit the replication of HIV. The HeLa immortal cell line was infected with the virus SCR-HIV (produced in HEK293T cells) and then treated with helium gas plasma at different doses. The quantity of the P24 antigen was detected by ELISA method in the supernatant and viability of cells were checked by XTT viability assay. The optimum voltage is 12 kV and exposure time is 240 s to obtain significant virus inhibition and toxicity to HeLa cells after plasma treatment. These outcomes showed that anti-HIV activity of nonthermal plasma and can affect both virus particles and infected cells and can be considered as potential treatment option against HIV diseases. Recently, nitrogen gas plasma exposure to influenza A and B viruses also showed the damage of viral genome and proteins, including nucleoprotein, hemagglutinin, and neuraminidase inactivation [239]. These modifications are due to changes in lipid content of viral envelope supported by Fourier-transformed infrared spectroscopy investigation and oxidation of viral protein content. The mechanism of this inactivation effect is based on heat, UV, and oxidative stress (long-lived hydrogen peroxide and other species) present in the plasma system. Mainly, oxidative stress appeared to be the important factor in the inactivation of influenza virus. Herpes Simplex Virus Type 1 (HSV-1) is another virus which can cause dreadful herpes keratitis in human cornea by replication in corneal epithelial cells. Recently, DBD plasma is used to treat HSV-1 viral load in corneal epithelial cells in the cornea [240]. The in vitro and ex vivo study indicated nonthermal DBD plasma highly reduced corneal HSV-1 infection without developing explicit toxicity. Adenoviruses are of more than 70 types and some of them can cause serious respiratory infections, gastrointestinal infections, or eye infections. Also, there is proper anti-adenovirus therapy is available in the clinics. Plasma treatment studies suggest that some specific type of adenovirus can be inactivated [241]. Inactivation is directly correlated by capsid protein oxidation by plasma treatment. Finally, cold plasma, a green or environmentally friendly technology, can be used as a tool to inactivate viruses. It can inactivate various human, animal, and plant viruses in various matrices [58]. For using cold plasma, it is important to tune correct parameters to properly inactivate viruses.

It is already shown that capsid protein and nucleic acids can be damaged by various RONS generated by the cold plasma. Up to now, only a few studies reported on plasma treatment can be correlated with virus reduction and inactivation. Recently, few reports indicated that plasma can be an effective tool to inactivate SARS-COV2 (COVID-19) virus [58–63]. Also, in vivo studies indicated no side effects with low invasiveness, simplicity, better wound healing, and inhibition of pain and inflammation [242]. Plasma medicine researchers could make devices which quickly and easily inactivate viruses present in hospital room and ambulances. These kinds of devices can sterilize both hard and soft surfaces and so can be game-changers for hospital facilities. It can be used to clean solid surfaces such as handphones, reusable masks, and other personal handheld devices. Further, quite a lot of new insights can be expected over the next few years related to an emerging virus such as COVID-19 (SARS-CoV-2) or other pathogenic viruses. Investigations in both cell culture models and patients must be initiated together to understand the antiviral activity of various cold plasma devices to treat COVID-19.

### Nonthermal plasma-based dental applications

Tissue engineering and regenerative medicine is the study that involves restoring and replacing the lost or damaged tissue which the interest towards it is ever increasing due to several reasons. In the field of dentistry, tissue engineering is very important as common diseases such as periodontal disease would result in both hard and soft tissue damage, which would then require “rebuild” of such structures either by use of biomaterials or through guided tissue regeneration. Also, a damaged tooth cannot regenerate while a lost tooth would require replacement using a dental implant. Tissue engineering following the periodontal disease would require the removal of pathogenic bacteria related to periodontal inflammation while aiding increased bioactivity by hard and soft tissue cells of periodontal tissues. Meanwhile, tissue engineering during the periodontal treatment would require materials such as scaffolds that would aid tissue regeneration. Also, placement of the dental implant in the alveolar bone would require prevention of bacterial attachment on the surface of the dental implant (that would otherwise result in disease commonly known as peri-implantitis) while aiding the attachment and growth of hard tissue cells on the surface (which would result in integration with the surrounding bone, which is commonly known as osseointegration).

There have been several attempts to improve tissue regeneration in dentistry by various methods. Recent works have highlighted that chemical modifications on either extracellular matrix or biomaterials may affect cellular activity. However, the complete mechanism of how the change in chemistry results in cellular activity alteration and the antibacterial result is still to be understood. Also, there is a limited method of changing the chemical nature of the extracellular matrix, which is often difficult to carry out. The change in the chemistry of the extracellular environment often would result in damage to a biomaterial or other biologically favorable properties.

Cold plasma can be applied to sterilized medical and dental equipment and materials. The application of plasma in dentistry sterilization is safe and cost-effective and one of the best alternatives to conventional methods. Recently, plasma is also used for dental tissue and stem cell treatment, dental-related implant biomaterial modification, and tooth whitening without having any damage to cells, tissues, and dental biomaterials. Cold plasma holds great potential such as for effective sterilization, microbial inactivation, and dental cavity treatment [243–245]. The gas plasma-based treatments are patient-friendly (especially for children and old aged people) and are painless and drill-less. Previous several investigations reported to treat dental pathogenic microbes related to dental cavity disorders such as *Streptococcus mutans* and *Staphylococcus aureus*, *Porphyromonas gingivalis*, and *Enterococcus faecalis* [246–248]. It is also reported that the plasma antimicrobial effect is more on Gram-negative than on Gram-positive bacteria [249]. Gummy smiles and gingival hyperpigmentation are very common dental cosmetic-related issues. Nonthermal plasma technology also can be used in both cases as well as soft tissue treatments in the future [250]. Also, cold plasma as a source of RONS can be used for dental stem cell applications for more fast bone formation and dental tissue healing with molecular expression related to growth factors [251, 252]. Remarkably, cold plasma is not only alternative therapy for dental treatment but also can be used for synergistic treatments for inactivation of microbes and inhibition of biofilms [244, 253–255]. Another synergistic application of nonthermal plasma is based on teeth bleaching together with a low concentration of bleaching agent to inhibit bacteria and biofilm formation [253, 256]. Plasma is greatly used for dental biomaterial treatments. Cold plasma exposure can have a potential application on the enhancement of dentin adhesion [257]. Cold plasma treatment for 30-s-increase nano hardness, Young’s modulus of hybrid layer, and hydrophilicity are responsible for better adhesive properties of biomaterials [258]. The plasma treatment on powder-injection molded zirconia implants increased hydrophilicity of the surface and osseointegration of the implants affecting topography [259]. Similar investigation suggested that cold plasma with UV treatment on titanium, zirconia, and modified polyetheretherketone surfaces improved the surface cytocompatibility of implants. Surface modification of dental substrates by nonthermal plasma indicated potential application in the dental application by changing surface and chemical properties [260]. Here, the possibilities of using such technology for dental application is considered, which could subsequently provide bases for novel dental medical devices. Market for dental medical devices is rapidly growing. As global aging of population and increasing interest towards better quality of life, the market related to dental medical devices such as the dental application of bio-plasma is expected to have global impact.

The objective of plasma medicine researchers is to develop nonthermal plasma technologies that can be used for dental applications. The characterized bio-plasma sources can be used on extracellular environments of dental cells/tissues for various future applications. Also, indirect treatments such as bio-plasma-treated dental biomaterials and plasma-activated liquid can provide an understanding of how modifications of the extracellular environment of dental cells/tissues would influence the bioactivity of related cells and pathogenic bacteria, with special reference to periodontitis. The investigation related to future potential and optimization industrial application of the bio-plasma is much needed for the preparation of the future dental medical device.

### Plasma application to agriculture

Plants in agriculture are most vulnerable to fungus. Thus, nonthermal atmospheric plasma was applied to fungi, to find the lethal effectiveness of plasma to fungi, which may attack the agricultural plants or foods. Filamentous fungi had been rarely explored in terms of plasma treatments. One literature [261] presents the cellular and molecular responses of the filamentous fungus *Neurospora crassa* to an argon plasma jet at atmospheric pressure. The viability and cell morphology of *N. crassa* spores exposed to plasma were both significantly reduced depending on the exposure time when treated in water. Microwave plasma at atmospheric pressure has been developed for surface modification, nanoparticle production, gas abatement, and biomedical applications including sterilization and detoxification. A plasma jet source at the atmospheric pressure was excited by 2.45 GHz microwaves and operated at a low energy regime with an average power of 0.8–1.6 W. This microwave plasma [262] was applied to examine fungal inactivation and find physical conditions of plasma (electrical power, pulse widths, and fed gases) at which the highest inhibition effects on fungal growth was achieved. Spore germination and hyphal growth of the fungus were dramatically decreased when oxygen was used in the plasma discharge, and this might be due to the elevation in the level of oxygen (O) radical. The level of O radical in the plasma generated from Ar and oxygen was also enhanced by the increased power and pulse width. Hyphal growth of the fungus was more inhibited when greater power or longer pulse was applied. It appears that plasma effects were varied among different fungal species. Different levels of inhibition on spore germination and growth of three filamentous fungi, *Neurospora crassa*, *Fusarium graminearum*, and *Fusarium oxysporum*, were observed.

Atmospheric pressure nonthermal plasma is an ionized gas in which various reactive species are produced. One literature [263] analyzed the influence of the ionic strength of surrounding solutions (environment) on the antimicrobial activity of plasma in relation to the plasma-generated reactive species using a model filamentous fungus, *Neurospora crassa*. Our data revealed that the presence of sodium chloride (NaCl) in the background solution attenuated the deleterious effects of plasma on germination, internal structure, and genomic DNA of fungal spores. These results suggest that the surrounding environment may affect the behavior of reactive species, which leads to different biological consequences regardless of their quantity. Moreover, the microbicidal effect of plasma can be synergistically regulated through control of the microenvironment. Nonthermal plasma treatment on fungal viability in a plant host was also carried out [264]. Reactive oxygen and nitrogen species can have either harmful or beneficial effects on biological systems depending on the dose administered and the species of the organism exposed, suggesting that application of reactive species can produce contradictory effects in disease control, pathogen inactivation, and activation of host resistance.

Seed sterilization is essential for preventing seed-borne fungal diseases. Previous literature [265] had examined antifungal activity of ozone and arc discharge plasma, potential tools for seed sterilization. Ozone inactivates *Fusarium fujikuroi* (fungus causing rice bakanae disease) spores submerged in water more efficiently than arc discharge plasma. However, fungal spores associated with or infecting rice seeds are more effectively deactivated by arc discharge plasma. ROS generated in water by ozone may function as a powerful fungicidal factor. On the other hand, shockwaves generated from arc discharge plasma may have greatly contributed to antifungal effects on fungus associated with rice seeds. In support of this notion, the addition of ultrasonic waves in ozone-generating water had greatly increased the efficiency of seed disinfection. Microbial decontamination of seeds by plasma had been also demonstrated [266]. Rice seeds infected by *Fusarium fujikuroi* (fungus causing bakanae disease) could be disinfected via underwater arc discharge plasma. Thus, the mechanism of disinfection and the effect on disease severity were further investigated. Also, disinfection of rice seeds in the air by surface DBD plasma was evaluated. A shockwave of 11 atm pressure was generated during arc discharge, which likely caused fungal detachment from the seed surface. Moreover, reactive oxygen species such as atomic oxygen (O) were emitted from the underwater arc discharge plasma and could have contributed to the degeneration of the chemical composition on the seed surface and the inactivation of the fungal spores. Plasma may enhance seed germination. The potentiality of nonthermal atmospheric pressure plasma in activating plant development had been evaluated [267]. The seed germination rate of *Coriander sativum*, a herbaceous plant with a slow germination rate, had been significantly increased over time compared to control after micro-DBD (dielectric barrier discharge) plasma treatment with N_2_ feeding gas.

## Conclusion

Plasma bioscience and medicine is a multidisciplinary field that combines physics, chemistry, engineering, bioscience, and medicine. In the past decade, this field has developed into an innovative and growing research field. This innovative field started in the 1990s with sterilization and decontamination applications of nonthermal plasmas, and it gradually expanded to eukaryotic cell treatment. The current application of nonthermal plasma in dermatology wound healing, dentistry, microbial inactivation, and cancer treatment aims to overcome current healthcare challenges. Plasma has the potential for cancer immunotherapy and can be applied to clinical translation. Tremendous efforts have been recently made focusing on the use of synergistic effects of nanomaterials and plasma towards cancer applications. Optimization of the therapeutic range of PAM and plasma is crucial because their sensitivities are different for different cell types. Plasma may also be used in food processing and agriculture applications, owing to its antimicrobial nature and its enhancement of the seed germination effect, respectively. The proficiencies of plasmas as a bactericide and fungicide improve the usefulness and competence of plasma treatments. Using ambient air as a process gas enables more cost-effective plasma industrial applications. Most importantly, nonthermal plasma was also proven to be safe and effective against SARS-COV-2 (COVID-19) by preventing transmission of infections; this may be a new opportunity to control or treat dreadful viral diseases. Present plasma-based healthcare studies are related to source design for specific uses and delivery strategies without affecting other cells or tissues, thereby maintaining safety standards relevant to the healthcare system. However, nonthermal plasma applications should not be limited to surface treatments. The development of endoscopic or small-sized plasma devices that can produce plasma inside the human body, particularly in internal tissue and organs, must be considered in the future. Moreover, in the future, studies should focus on understanding the mechanism of action and safety for plasma biological applications, both in laboratories and clinical practice. Further international standards for plasma devices and their specific application should be made with the cooperation of plasma researchers worldwide. With this development, it is possible to satisfy the high expectations of patients and the safety required of plasma devices. In conclusion, nonthermal plasma can be used for biological and clinical applications with a low treatment cost, minimal invasiveness, and no side effects.
